# Health for all? A cost-utility evaluation of Colombia's policy to enroll Venezuelan migrants (2021–2023)

**DOI:** 10.1016/j.jmh.2025.100374

**Published:** 2025-10-31

**Authors:** Oscar Espinosa, Paul Rodríguez, Valeria Bejarano, Santiago León, José Luis Ortiz

**Affiliations:** a Economic Models and Quantitative Methods Research Group, Universidad Nacional de Colombia, Bogotá, D.C., Colombia; b School of Economics, Universidad del Rosario, Bogotá, D.C., Colombia; c Inter-American Development Bank. Bogotá, D.C., Colombia

**Keywords:** Universal health coverage, Subsidized health insurance, Health system evaluation, Cost-utility analysis, Public policy, Colombia, Venezuelan migrants

## Abstract

**Introduction:**

From 2021 to 2023, Colombia implemented a national policy to expand health insurance coverage for over 1.4 million Venezuelan migrants through its subsidized health insurance system, which provides universal health coverage to low-income Colombian populations. This study evaluates the cost-utility of the intervention, focusing on its health and economic returns.

**Methods:**

We conducted a cost-utility analysis by translating government expenditure per enrolled migrant into health outcomes (QALYs/YLLs) using Colombia-specific cost-effectiveness thresholds. Health benefits were measured in quality-adjusted life years (QALYs) gained and years of life lost (YLLs) averted. Colombia-specific cost-effectiveness thresholds (CETs) were used to estimate outcomes, and an internal rate of return (IRR) analysis assessed the program's social profitability.

**Results:**

The intervention generated an estimated 10,259 QALYs gained or 11,843 YLLs averted between 2021 and 2023. The IRR reached 27.5% when QALYs were valued at 100% of Gross Domestic Product per capita, indicating strong social returns. Women and adults aged 19–44 showed the greatest health gains, with urban areas receiving the highest benefits. This evidence demonstrates that even health policies can yield high social returns, providing a model for other countries navigating large-scale migration.

**Discussion:**

Expanding subsidized health insurance to migrants proves to be a highly cost-effective policy. This analysis supports Colombia’s approach as a scalable and impactful model for inclusive public health aligned with universal health coverage goals.

## 1 Introduction

The integration of migrants into national social protection systems remains one of the most complex and politically sensitive challenges for countries experiencing large-scale human mobility. While growing international evidence highlights the benefits of including migrants in health systems—such as reducing inequities, preventing public health crises, and enhancing labor productivity (Onarheim et al., 2018, Matlin et al., 2018, Garfield and Orgera, 2019, Jalali, 2015, Sarría-Santamera et al., 2016)—governments often hesitate to adopt inclusive policies, citing concerns over fiscal burden and political resistance.

Colombia offers a compelling counterexample. By early 2024, it had received over 2.8 million Venezuelan migrants (Licheri et al., 2024), many of whom were living in conditions of vulnerability and without access to essential public services. Rather than limiting their access, the Colombian government undertook a comprehensive inclusion strategy. Between 2021 and 2023, with support from international cooperation, it extended subsidized health insurance coverage to more than 1.4 million Venezuelans (Banco Interamericano de Desarrollo 2020, Ministerio de Salud y Protección Social 2024), operationalizing a version of universal health coverage (UHC) rarely seen in Latin America.

Colombia’s General Social Security Health System (GSSHS), created by Law 100 of 1993 (Ministerio de Salud 1993), is an insurance-based model grounded in structured pluralism (Londoño and Frenk, 1997, Ministerio de Salud y Protección Social 2007). It includes three enrollment schemes: contributory (for formal workers), subsidized (for low-income individuals, currently covering 26.5 million people), and special schemes for specific public employees (Melo-Becerra et al., 2023). Health benefits are financed through a per-capita payment (Capitation Payment Unit, CPU), adjusted for demographic and geographic criteria. Over 90% of approved drugs and procedures are covered under the Health Benefits Plan (HBP-CPU) (Ministerio de Salud y Protección Social 2022), with other technologies financed through supplementary mechanisms. Health care providers include both public and private entities, with a higher prevalence of private providers managing public health funds in urban areas. Meanwhile, territorial governments are responsible for coordinating and managing public health interventions.

The GSSHS has achieved near-universal health coverage (98.6% by mid-2024), low out-of-pocket expenditure (13.7% in 2021), and significant gains in infant mortality, vaccination coverage, and waiting times—comparable to Organization for Economic Co-operation and Development (OECD) benchmarks (Ministerio de Salud y Protección Social 2024, Ministerio de Salud y Protección Social 2023, Ministerio de Salud y Protección Social 2023, Organisation for Economic Cooperation and Development 2020, Pan American Health Organization 2024). Yet, persistent challenges remain: geographic inequities, weak primary care, high maternal mortality, and increasing financial pressures from demographic and epidemiological shifts—including large-scale migration (Mora-Moreo et al., 2023, Pinto et al., 2018). These challenges stem from insufficient health governance at the territorial level, which limits the adaptation of services to reach the most vulnerable subpopulations. Additionally, there is a lack of innovation in insular, rural, and dispersed areas, as well as inadequate adaptation of primary healthcare services in both private and predominantly public settings to the identities, needs, and circumstances of diverse vulnerable groups.

The Venezuelan immigration has placed additional strain on the GSSHS due to increased demand for healthcare services provided to this population. Since 2010, migration of Venezuelans to Colombia has increased considerably. As of January 2024, Colombia has received more than 2.8 million Venezuelans (Licheri et al., 2024). The migrants health needs prior to 2021 were largely provided through emergency services, leading to a high cost of care (much higher than that of the enrolled population) and a deterioration of the public health situation of the host communities (Prada et al., 2023). In this context, one of the main health inequity gaps was the lack of insurance coverage for this population. At the end of the last decade, only 11% of the immigrant population had been enrolled with the GSSHS, 62.6% in the contributory scheme and 37.4% in the subsidized scheme. This was due to regulatory barriers in the registration systems, lack of knowledge of the processes and enrolment routes, and/or cultural issues (Banco Interamericano de Desarrollo 2020). Also, by that time, a total of 3,931,799 healthcare services were provided to almost half a million Venezuelan immigrants, of which 21% were pregnant women requiring obstetric care. For this reason, funding requests to the national government exceeded 50 million dollars (Banco Interamericano de Desarrollo 2020).

Since 2017, the Colombian government has progressively implemented policies to expand health coverage for Venezuelan migrants. Key milestones include recognizing the Special Permit of Permanence (SPP) as a valid document for health system enrollment (Resolution 3015 of 2017), prioritizing service delivery and enrollment strategies through CONPES 3950 (2018), and guaranteeing coverage for children of Venezuelan parents born in Colombia (Resolution 8470 of 2019). Decree 064 of 2020 further prioritized enrollment for uninsured vulnerable groups, including migrants with SPP. Most recently, CONPES 4100 (2022) framed the long-term socioeconomic integration of migrants as a national development strategy. Together, these efforts represent a comprehensive legal and policy framework to support health inclusion for the Venezuelan migrant population. In this context, the Government of Colombia, supported by a multilateral development bank loan and a grant program funded by the German government (Ministerio de Salud y Protección Social 2024), implemented various strategies to increase health insurance coverage among Venezuelan migrants and to ensure payment of obstetric health services for this population (https://www.iadb.org/en/project/CO-L1248).

This study addresses a critical evidence gap regarding the health benefits and economic return of integrating vulnerable migrant populations into Colombia’s health system. Despite recent progress in expanding coverage, few analyses have quantified health outcomes using standardized metrics. By applying a cost-utility approach, we provide a rigorous and timely assessment—particularly relevant as Colombia faces growing fiscal pressures and requires evidence-based strategies to support sustainable health policy decisions.

This paper presents a cost-utility analysis of this policy intervention. It estimates health benefits in terms of quality-adjusted life years (QALYs) gained and years of life lost (YLLs) averted and compares these to program costs. The study contributes to global debates on the fiscal and social returns of migrant integration and offers empirical evidence for designing equitable and sustainable health policies in middle-income countries.

## 2 Methodology

To calculate the cost-utility ratio of the interventions under the immigrant health policy, the basic information on the loan program being analyzed is presented below. First, the monetary disbursements-which began in August 2021-are detailed in Table 1.

**Table 1:** Investments by activity, 2021-2023.

Investment by activity/year (USD Millions)				
Activity	2021	2022	2023	Total
1. Insurance coverage for immigrant population	25	25	-	50
2. Financing of obstetric health care for vulnerable immigrant population not enrolled to the GSSHS	-	5.862	5.863	11.725
**Total invested**	**25**	**30.862**	**5.863**	**61.725**

Second, Table 2 presents the baseline for each of the two subcomponents of the study, along with the proposed and achieved targets, according to the reports of the GSSHS governing body (Banco Interamericano de Desarrollo 2020).

**Table 2:** Results expected and achieved by the health system 2021-2023.

Variables	Results by activity/year	Baseline (2019)	2021	2022	2023	Cumulative 2021 to 2023
Migrants enrolled to the GSSHS (accumulated)	Expected	115,928	220,000	320,000	425,000	965,000
	Reached	-	463,433	693,047	261,358	1,417,838
Obstetric Healthcare services for migrant population	Expected	0	-	9,500	9,500	19,000
	Reached	-	-	9,602	15,339	24,941

Following the guidelines established by Drummond et al. (2015) and Gray et al. (2010) for conducting cost-utility analysis in the field of health economics, we proceed to explain the perspective of the study, the estimation of costs, benefits, discount rates and cost-utility analysis itself.

This is a cost-utility study, given the ability to estimate the intervention costs and link them to health benefit measures that incorporate, for example, quality of life. The benefit measures used are QALYs^1^ gained and YLLs^2^ averted. For this kind of analysis, it is common to use individual-level data where detailed information on both costs and health outcomes is available for patients who receive treatment and those who do not. However, in this study, information on the costs and benefits for individuals who do not receive treatment is not available. For this reason, two fundamental assumptions are required for methodological development:

I. The resources invested by the government were pooled and distributed according to the demographic structure of the migrant population.
II. The costs and benefits experienced by the beneficiary population are assumed to be similar to those experienced by the Colombian population within the health system.

**Fig. 1:**
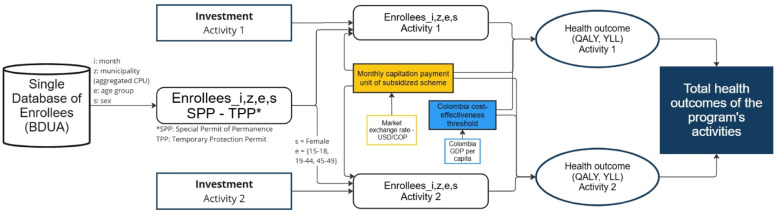
Schematic of analytical methodology.

These assumptions imply certain restrictions in the implementation, which will be discussed in detail, and require an alternative methodological approach. Fig. 1 is provided as a guide to help understand the role of each step in the estimation of health outcomes.

For this section we treat costs as given, referring to the investment of the government in terms of CPU. Unfortunately, since we do not have access to the anonymized microdata to identify which individuals were enrolled through the loan program, we assume that enrollment was distributed proportionally across the migrant population throughout the country, by sex, municipality and age group. This corresponds to the first step in Fig. 1, where we calculate the number of enrolled migrants represented by the variables $Enrollees_{i,z,e,s}$ (where i is the month, z the geographic area, e the age group and s the sex). In this sense, we are determining the number of beneficiary members, given that we know the total value of the intervention’s costs.

Information on Venezuelan immigrants enrolled with the subsidized scheme of the GSSHS disaggregated by month, year, age, municipality and sex is extracted from the Single Database of Enrollees (BDUA). The monetary value of the CPU of the subsidized scheme segmented by year^3^, sex, age and geographic area are taken from resolutions 2503 of 2020 (Ministerio de Salud y Protección Social 2020), 2381 of 2021 (Ministerio de Salud y Protección Social 2021) and 2809 of 2022 (Ministerio de Salud y Protección Social 2022) of the Ministry of Health and Social Protection (MHSP).

To convert CPU magnitudes into U.S. dollars (USD), the average daily market exchange rate for each year is used, as reported by the Central Bank (Banco de la República 2024). Price deflation is performed using the consumer price index computed by the National Administrative Department of Statistics, taking as the base year 2021 (Departamento Administrativo Nacional de Estadística 2024).

### 2.1 Estimating health benefits

In the absence of a control group, health benefits (QALYs/YLLs) were estimated by dividing the total expenditure per enrolled migrant by a Colombia-specific cost-effectiveness threshold (CET), which represents the average cost of generating one unit of health outcome within the system.

To estimate health benefits, represented on the right-hand side of Fig. 1, the main methodological challenge is approximating the counterfactual scenario of non-participation in the health system. The difficulty lies in the absence of a natural control group, due to the universal nature of the Colombian health system. Additionally, there were no administrative records for individuals excluded from the system. However, the CET provides a statistical measure of efficiency that reflects how monetary investments translate into health outcomes. Its use is justified by its ability to express the level of expenditure in the health system necessary to obtain a health outcome measure.

In this sense, the health gains of the population estimated in the previous step ($Enrollees_{i,z,e,s}$) are quantified using the value transferred per person and translating these resources into outcomes through the CET. The use of the threshold relies on the assumption of similar levels of access and patterns of use of the system between the Colombian and Venezuelan populations. Moreover, it generalizes the interpretation of the threshold that is associated with marginal increases rather than full inclusion in the health system. The implications of these assumptions are addressed in the discussion section.

For example, for a single migrant enrolled in 2022, the health benefit is calculated by dividing the monthly government expenditure per enrollee (CPU) specific to their demographic group by the CET for that year. This yields the estimated health gain (in QALYs or YLLs) attributed to that individual. The overall health benefit is then obtained by summing this calculation across all enrolled migrants, with future gains discounted to present value. Given that we have the specific investment data for the different years (from August 2021 to December 2023) we will calculate the health outcomes measured in QALYs and YLLs, as follows:

(1) $$HO_{Program\;}=\sum_{i=8}^{12}Enrollees_{i,z,e,s,2021}*CPU\_SS\_M_{z,e,s,2021}/CET_{HO,2021}+\frac{1}{1+\delta }\;\sum_{i=1}^{12}Enrollees_{i,z,e,s,2022}*CPU\_SS\_M_{z,e,s,2022}/CET_{HO,2022}+{\left(\frac{1}{1+\delta }\right)}^{2}\sum_{i=1}^{12}Enrollees_{i,z,e,s,2023}*CPU\_SS\_M_{z,e,s,2023}/CET_{HO,2023},$$

whereEnrollees are the enrolled immigrants to the subsidized scheme of the GSSHS, CPU_SS_M is the monthly CPU of the subsidized scheme, HO={QALY,YLL} is defined as the health outcome measure,i={1,…,12} is the month of the year of study,CET is the cost-effectiveness threshold for Colombia (understood as the monetary value that produces one QALY gained or one YLL averted),z is the municipality according to the geographical area of the CPU),e={≤1,1−4,5−14,15−18,19−44,45−49,50−54,55−59,60−64,65−69,70−74,≥75} is the age group established for the CPU, and s={Female,Male} is the sex. δ corresponds to the discount rate^4^.

On the other hand, the CET per QALY and per YLL is taken from recent research by Espinosa et al. (2022), in which these parameters were estimated using real-world evidence and robust econometric methods^5^ (Instituto de Evaluación Tecnológica en Salud, 2025). For the current case study, the CET per QALY is 86% of Gross Domestic Product per capita (GDPpc)^6^ and the CET per YLL is 74.5% of GDPpc^7^ for the respective year of analysis (Instituto de Evaluación Tecnológica en Salud, 2025)^8^.

Equation [1] is subject to two conditions. First, a budget constraint of USD 61.725 million over the 29 months duration of the program (beginning in August 2021). Second, individual obstetrics care^9^ was implemented in all territorial entities of the country, except for the municipalities in the departments of Amazonas, Caldas, Córdoba, Guaviare, Huila, San Andrés and Providencia, and Vaupés. This, in accordance with resolutions 1383 of 2021, 1792 of 2021, 1832 of 2021, 2130 of 2021, 2490 of 2022, 2205 of 2022, 2683 of 2022 and 2025 of 2023 issued by the MHSP (Ministerio de Salud y Protección Social 2024).

To evaluate the cost-effectiveness of the program, the internal rate of return (IRR) methodology was used, which monetizes health benefits (measured in QALYs and YLLs) relative to the investment made. This methodology makes it possible to determine the economic performance of the program by considering the value of the health benefits as a percentage of GDPpc, thus providing an estimate of its long-term economic and social impact.

Estimating the IRR requires monetizing health gains using a 'price' for a unit of health outcome, e.g., QALYs or YLLs. The IRR is then estimated across various monetization scenarios for QALYs and YLLs. In the case of QALYs, the literature usually uses CET (see for example (Glover et al., 2014))^10^, while the value of a disability-adjusted life year is commonly priced with the statistical value of a life (SVL) (Robinson et al., 2019a, Robinson et al., 2019b).

Several studies in the literature estimate the SVL for Colombia, using different methodological approaches. The first reference value was established by Viscusi and Masterman (2017), at 1.228 million USD of 2015 (1.185 of 2021)^11^. Mardones and Riquelme (2022) established one of 0.78 million USD (0.78 of 2021). Sweis (2022), estimated it between 0.7 and 3.6 million USD in 2020. Vasquez-Lavin et al. (2022) estimated it between 0.95 and 1.54 million USD (between 1.01 and 1.63 of 2021) for the year 2017. Consequently, estimates generally range from 0.78 to 1.63 million USD in 2021. Given that life expectancy in Colombia, according to *World Population Prospects* was 77 years as of 2019^12^, the value of one year of life (unadjusted for quality) would range from 10.13 (162% of GDPpc) and 21.17 thousand USD (339% of GDPpc).

## 3 Results

### 3.1 Enrollment

**Fig. 2:**
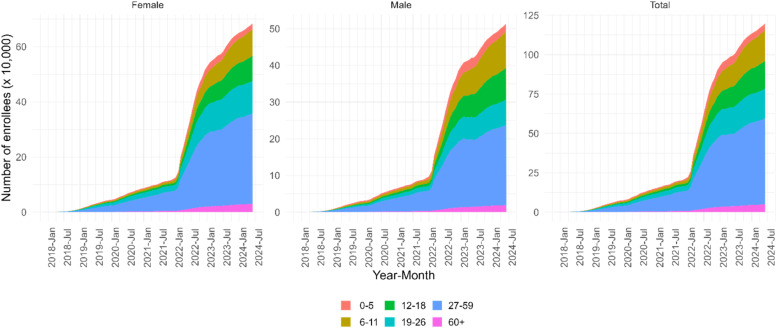
Enrollees with Special Permit of Permanence (SPP) or Temporary Protection Permit (TPP), 2018-2024.

**Note:** enrollees with 'Special Permit to Stay (SPP)' and 'Temporary Protection Permit (TPP)' were taken, including the status 'active', 'active due to emergency', 'labor protection', 'suspended', 'suspended due to document' or 'suspended due to arrears'.

Within this legal framework and acknowledging that most Venezuelans in Colombia live in vulnerable conditions, a large percentage is enrolled with the subsidized scheme in the GSSHS (71.2% in 2023), reaching an average of 1,020,290 people in 2023 (see Fig. 2). According to BDUA, 2022 saw a notable increase in enrolment to this scheme, rising from an average of 181,781 people in 2021 to 581,539, and nearly doubling by 2023. As of June 2024, the age distribution of enrollees by life cycle was as follows: early childhood 3.48%, infancy 16.34%, adolescence 14.82%, youth 15.62%, adulthood 45.59% and old age 4.16%. In addition, there is a higher proportion of women (57%) compared to men (43%).

In 2021, Venezuelans enrolled in the GSSHS received more healthcare services per person and at a higher cost than Colombians, conditional on age group and sex (more than 30%, averaged across various socio-demographic categories) (see Supplement 1). This is consistent with the expectation that pre-policy barriers generated a selection effect, where only the sickest migrants accessed medical care. However, in 2022 and 2023, utilization rates are slightly lower for Venezuelans than for Colombians (about 8%), and costs slightly higher (Supplement 1). These findings suggest that utilization patterns are currently comparable and that effective enrollment levels obtained are probably comparable between both populations.

The mortality profile of Venezuelan migrants between 2012 and 2022 reflects the consequences of delayed or absent access to health services prior to their formal integration into Colombia’s health system (see Supplement 2). The predominance of deaths from HIV/AIDS, tuberculosis, and obstetric complications—particularly among men and pregnant women—underscores the historical lack of preventive and primary care for this population. The spike in COVID-19 mortality in 2021 and its sharp decline in 2022 illustrate both the health vulnerability of migrants and the responsiveness of the health system once access is granted. Complementary service utilization data from 2021 to 2023 show a marked increase in healthcare access, particularly for maternal and chronic conditions such as hypertension, cancer, and diabetes. These trends suggest that the inclusion of migrants in the subsidized scheme not only addressed an urgent public health gap but also enabled a shift toward more regular and preventive care.

### 3.2 Costs

**Fig. 3:**
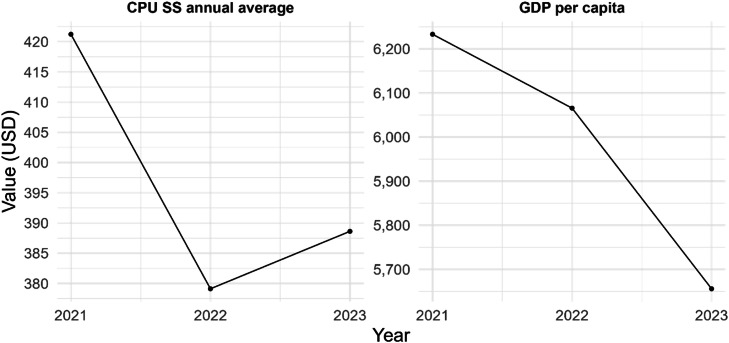
CPU of the subsidized scheme and GDP per capita, Colombia, 2021-2023 (constant prices, 2021).

For the years 2021, 2022 and 2023 the GDPpc was USD 6,233, USD 6,066 and USD 5,656, respectively, reflecting an average rate of change of -4.7%. Regarding the annual CPU of the subsidized scheme, it averaged USD 421 in 2021, USD 379 in 2022 and USD 389 in 2023, having an average rate of variation of -3.8%, values in constant 2021 prices (see Fig. 3).

According to the assumptions presented previously, Table 3 shows the total cost for the different age groups of interest by sex (constant prices 2021).

In general, women represent higher costs for the health system, although men stand out in 19 to 44 years of age. Between the ages of 1 and 14, costs are similar, but from this age onwards disparities begin to appear, being higher for women between the ages of 19 and 49. After the age of 50, the average cost for women is approximately 54% higher than for men.

Applying the methodology developed in the previous section and using the base scenario with a 3% discount rate, it is found that the estimated benefits generated for Colombia, in terms of health outcomes, are 10,259 QALYs gained or 11,843 YLLs averted (Table 4). By sex and age group, women registered greater benefit, with 7,346 QALYs or 8,479 YLLs, compared to men, who achieved 2,914 QALYs or 3,363 YLLs. In addition, the 19-44 age group, which has the largest number of beneficiaries, obtained 6,528 QALYs or 7,536 YLLs, followed by the 45-49 age group, with 713 QALYs or 823 YLLs and the 5-14 age group with 633 QALYs or 730 YLLs.

**Table 3:** Total cost 2021-2023 (USD) of subcomponents by age group and sex, constant prices 2021.

Age group	Male	Female
<1	761	501
1-4	638,033	609,458
5-14	1,687,360	1,643,277
15-18	555,565	1,596,231
19-44	7,687,675	26,792,104
45-49	1,204,155	2,563,206
50-54	1,106,328	1,640,374
55-59	871,691	1,351,810
60-64	689,192	1,061,665
65-69	471,986	766,857
70-74	285,658	457,924
≥75	200,304	287,374

**Table 4:** Quality-adjusted life years gained, or years of life lost avoided due to the loan program, Colombia, 2021-2023.

Sex	Age group	QALY			YLL		
		Lower limit	Point estimate	Upper limit	Lower limit	Point estimate	Upper limit
Male	<1	0.08	0.14	0.57	0.10	0.17	0.63
	1-4	69.10	120.60	472.81	80.25	139.22	524.81
	5-14	183.60	320.45	1,256.30	213.22	369.92	1,394.46
	15-18	60.48	105.55	413.81	70.23	121.85	459.32
	19-44	832.62	1,453.25	5,697.31	966.95	1,677.58	6,323.86
	45-49	130.37	227.56	892.11	151.41	262.68	990.21
	50-54	119.82	209.13	819.88	139.15	241.41	910.04
	55-59	94.48	164.91	646.51	109.72	190.36	717.60
	60-64	74.77	130.50	511.62	86.83	150.65	567.89
	65-69	51.23	89.43	350.58	59.50	103.23	389.14
	70-74	31.03	54.16	212.34	36.04	62.52	235.69
	≥75	21.72	37.91	148.62	25.22	43.76	164.97
	Total	1,669.30	2,913.60	11,422.45	1,938.62	3,363.35	12,678.61
Female	<1	0.06	0.10	0.38	0.06	0.11	0.42
	1-4	65.99	115.17	451.53	76.63	132.95	501.18
	5-14	178.82	312.11	1,223.61	207.67	360.29	1,358.17
	15-18	173.94	303.59	1,190.19	202.00	350.45	1,321.07
	19-44	2,907.55	5,074.83	19,895.35	3,376.63	5,858.20	22,083.28
	45-49	278.19	485.55	1,903.55	323.07	560.50	2,112.89
	50-54	177.88	310.47	1,217.17	206.58	358.40	1,351.02
	55-59	146.66	255.97	1,003.52	170.32	295.49	1,113.88
	60-64	115.28	201.20	788.80	133.88	232.26	875.55
	65-69	83.25	145.31	569.66	96.68	167.74	632.31
	70-74	49.74	86.82	340.39	57.77	100.23	377.82
	≥75	31.18	54.43	213.38	36.21	62.83	236.85
	Total	4,208.54	7,345.57	28,797.52	4,887.51	8,479.45	31,964.45
Aggregate total		5,877.84	10,259.17	40,219.98	6,826.12	11,842.80	44,643.06

The financing of obstetric health care for the vulnerable immigrant population benefited more than 18,000 women, achieving outcomes of 1,909 QALYs or 2,204 YLLs, values that overcome the limitations of this population in terms of timeliness and quality of the health system, as well as the complexity of bureaucratic procedures that hinder access to care (Giraldo et al., 2021).

**Fig. 4:**
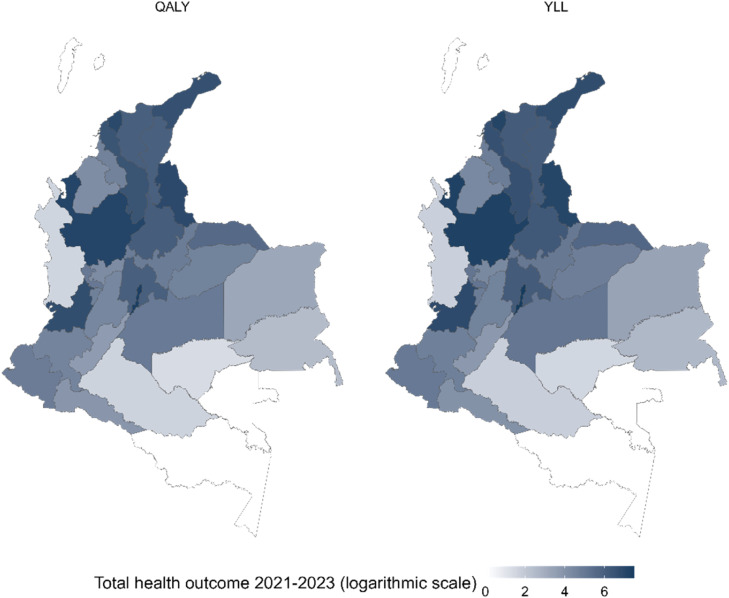
Health outcomes by department in Colombia 2021-2023.

**Note:** Values between 0 and 1 were kept at zero for the logarithmic scale.

Under the established assumptions, the estimated health benefits at the departmental level are shown in Fig. 4, while municipal-level values are detailed in Supplement 3. The capital district of Colombia stands out with the highest QALYs or YLLs, followed by the departments of Antioquia, Atlántico, Norte de Santander, Valle del Cauca, and La Guajira, all with values above 640 QALYs or 740 YLLs. These areas host the largest concentrations of immigrants (Rossiasco and de Narváez, 2023). When analyzed by CPU geographic zones, the regions with the greatest impact end up being the cities and suburbs (6,891 QALYs; 7,955 YLLs) and normal (2,345 QALYs; 2,707 YLLs), in contrast to the special zones (1,023 QALYs; 1,180 YLLs) and remote areas (0.34 QALYs; 0.39 YLLs), which have lower values.

Following the methodological approach, it was determined that when health outcomes are measured in QALYs, the point estimate of the cost-utility ratios were 10,259, 10,654, 9,894, 9,556 and 9,241, corresponding to costs of USD 52,204,711, USD 54,169,488, USD 50,386,344, USD 48,699,470 and USD 47,131,070 for the discount rates of 3%, 0%, 6%, 9% and 12%, respectively. On the other hand, when health outcomes are defined in YLLs averted, the cost-utility ratios were 11,843 at a 3% discount rate, 12,298 at 0%, 11,421 at 6%, 11,031 at 9% and 10,667 at 12% with the same respective costs. Table 5 summarizes the results described above for the study period.

**Table 5:** Cost-utility ratios with respect to (a) QALYs gained or (b) YLLs averted, for different discount rates, period 2021-2023.

Health outcome	Discount rate 3%.		0% discount rate		Discount rate 6%.		Discount rate 9%.		Discount rate 12%.	
	Point estimate HO	Total cost (USD)	Point estimate HO	Total cost (USD)	Point estimate HO	Total cost (USD)	Point estimate HO	Total cost (USD)	Point estimate HO	Total cost (USD)
(a) QALYs gained	10,259.17	52,204,711	10,653.86	54,169,488	9,894.06	50,386,344	9,555.50	48,699,470	9,240.85	47,131,070
(b) YLLs averted	11,842.80		12,298.41		11,421.33		11,030.51		10,667.29	

The health situation experienced by Venezuelan immigrants has highlighted the urgent need to guarantee access to health services for this population (Cubides et al., 2022, Doocy et al., 2019). The results following their integration into the national public health service systems have demonstrated a rapid attainment of the development benefits associated with social inclusion (Rossiasco and de Narváez, 2023). Supported by international cooperation, the strategy implemented by Colombia has enabled a better follow-up of chronic diseases that previously could not have been controlled on a regular basis (Cubides et al., 2022), thus improving the quality of life of the people who are now part of the GSSHS.

In 2022, Colombia’s health care system ranked 39th out of 94 countries, according to the composite index of health outcomes, surpassing several countries in the Americas (Giraldo et al., 2022). This system has benefited thousands of immigrants through coverage and access to more than ten thousand procedures and over one thousand active pharmaceutical ingredients included in the health benefits plan. Among the most frequent procedures during the analyzed period were first-time, control or follow-up and emergency consultations in general medicine and first-time dental consultations (with over 220,000 visits), as well as IV hemogram, urinalysis, serum creatinine, serum glucose, total cholesterol, triglycerides and high cholesterol tests, triglycerides and high density cholesterol (with over 142,000 visits), along with oral medicine, supragingival scaling, dental plaque control, dental obturation with light-curing resin and general dentistry control consultation (with over 132,000 visits).

Moreover, individual health education provided by general medicine and HIV 1 and 2 antibodies had more than 132,000 visits, according to Individual Health Services Provision Records (RIPS). The GSSHS has also expanded access to sexual and reproductive services, which previously had numerous deficiencies (Rivillas-García et al., 2021), as well as to epidemiological surveillance, emergency services, child health, and mental health, among others (USAID 2020).

Even with the multiple benefits, the current challenges of the system, exacerbated by the economic pressure derived from the regularization of the Venezuelan migrant population, are affecting the fiscal sustainability of the country (Granger et al., 2023). Although international cooperation, has facilitated the enrollment of more than 90,000 Venezuelans, it is important to note that sustaining this health policy to guarantee care for the entire migrant population (estimated at 2,813,997 by April 2024) will require a substantial investment effort.

### 3.3 Internal rate of return

**Fig. 5:**
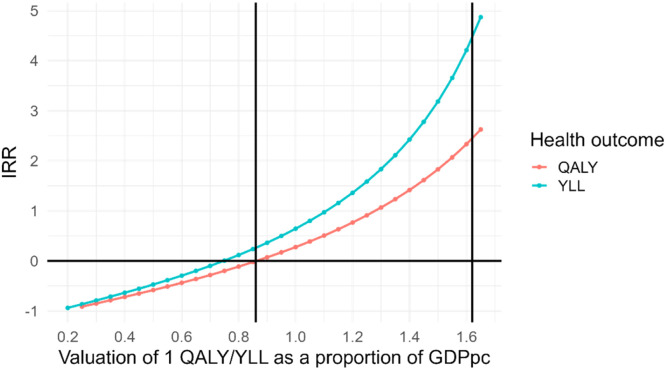
Internal rate of return (IRR) according to different monetization of QALYs and total YLLs.

**Note:** The calculation considers a flow net for each of the three years (2021, 2022, 2023). It is assumed that health gains start to be seen in the first year and that the value of the credit spread over the three years 'cash'. Each QALY/YLL is monetized as a percentage of GDPpc for the year. The first vertical line is at 0.86 (the threshold for QALYs), and the second vertical line is at 1.62 (Mardones and Riquelme's estimate of 1 SVL (Mardones and Riquelme, 2022)).

Finally, Fig. 5 illustrates how the IRR varies when the value of a QALY is expressed as a percentage of GDPpc, within the CET range of 21.9% to 150.1%. Valuing each QALY at 100% of GDPpc —a common assumption for opportunity‐cost pricing—yields an IRR of 27.5%. When YLLs are similarly valued at 100% of GDPpc, the IRR rises to 64%. Using the SVL instead—applying the most conservative SVL estimate of 1.62 GDPpc per YLL—raises the IRR to 445%. Across this broad range of benefit valuations, the program remains clearly socially profitable.

It is important to highlight that the confidence intervals for the CET, based on Espinosa et al. (2022), are wide, reflecting substantial uncertainty in converting cost per unit into QALYs and YLLs. While point estimates are used in the main analysis, this variability is accounted for in the IRR sensitivity analysis, providing a more comprehensive view of the range of possible outcomes.

## 4 Discussion

International evidence suggests that enrolling immigrants into formal health care systems prevents the deterioration of their health conditions, reduces barriers to care, and increases national labor productivity by supporting a healthier working population (Onarheim et al., 2018, Matlin et al., 2018, Garfield and Orgera, 2019, Jalali, 2015, Sarría-Santamera et al., 2016). At the beginning of this decade, the Colombian government implemented different initiatives to improve, among other aspects, the effectiveness and efficiency of its health policy for Venezuelan immigrants, which should be comprehensive and sustainable through their enrollment to the GSSHS. This study shows an IRR of 27.5% on this investment; a high value resulting from a shift from basic protection - vital emergencies - to full access to a comprehensive health benefits plan with remarkable health outcomes, particularly in urban areas.

Non-regularized migrants face adverse conditions in nearly all aspects of their lives. In particular, their health is compromised by limited access to health policies -particularly health insurance- and insufficient income to directly purchase healthcare services (Matlin et al., 2018). This runs counter to Target 3.8 of the Sustainable Development Goals (SDGs), which calls for UHC. Moreover, even if they are entitled to certain services, many migrants may not access them due to limited knowledge about navigating a foreign health system (Giraldo et al., 2021). Hence, there is a need to invest in more cost-effective health services, as well as to be more efficient in the management of the system's resources to strengthen health promotion and prevention services, both of which are necessary to improve morbidity and mortality indicators.

The role of migrants' regularization on their health outcomes has been scarcely explored in international literature. Most existing research refers to labor market outcomes, where greater formalization leads to better jobs, improving migrants' incomes. In the case of health, studies tend to focus on pricing the cost of regularizing migrants (Lu et al., 2020, Tschirhart et al., 2017, Xiong et al., 2017, Ferrara, 2020). Most of the research centered on Europe or North America, such as the cases of Spain (Elias et al., 2024), Italy (Deiana et al., 2021, Deiana et al., 2022), and the United States (Kossoudji and Cobb-Clark, 2002, Bratsberg et al., 2002, USAID 2006). The role of health and migration is mainly linked to the spread of infectious diseases (Doocy et al., 2019, Montalvo and Reynal-Querol, 2007, Baez, 2011, Ibáñez et al., 2021), and fertility (Amuedo-Dorantes et al., 2023, Avitabile et al., 2014, Amuedo-Dorantes et al., 2023, Salmasi and Pieroni, 2015).

Current evidence for Colombia supports similar results. Ibáñez et al. (2022), focusing on Venezuelan migrants, found that the 2018 regularization program, which granted the SPP, had strong benefits in terms of overall well-being, including income, consumption, and self-perceived physical and mental health (mobility, anxiety and depression, daily activities, self-care, pain fatigue, and health perception). However, this study’s limitation lies in the variation associated with the marginal socioeconomic conditions that determine eligibility for the subsidized health care system, which are difficult to extrapolate for individuals with high levels of poverty and who are the bulk of the population to be analyzed. In addition, the sample size was small, making it difficult to study measurable health outcomes of QALYs or YLLs. Even so, MHSP statistics confirm that health coverage for immigrants has improved through the strategy of enrolling Venezuelan migrants in the subsidized scheme, including newborns, children of parents not enrolled with the GSSHS. However, the implementation of actions to disseminate information on enrolment pathways and access still needs to be strengthened to health services.

A major challenge in evaluating the impact of regularization programs on the health status of migrants lies in the availability of data. Migrants are largely ‘in the shadows’ of administrative records, precisely because of the same barriers that prevent them from accessing. With this idea in mind, this research adopts an approximation based on the assumption that the gain a migrant receives from being included in the Colombian health system is equal to that of a Colombian: 1 QALY for every 86% of GDPpc invested in the health sector. Under this assumption, estimating the impact on health requires information on the number of enrollees and the variables that determine the expenditure required to insure them (place of residence, sex, and age).

Although the CET approach provides a useful benchmark for evaluating health interventions, it has been subject to criticism in the literature. CET-based decisions can oversimplify complex health system trade-offs by focusing narrowly on efficiency rather than broader societal values. In this context, while CET serves as a practical tool for this study, policy decisions should also consider other factors such as equity, feasibility, and the broader social impact of interventions, especially when dealing with vulnerable populations such as migrants.

This approach has several limitations that may affect the estimated impact, mostly leading to a conservative bias. First, an ‘income effect’ linked to labor regularization could improve migrants’ health independently of healthcare access, implying our results may underestimate the true policy impact. This underestimation may be amplified by the generally poorer baseline health of migrants compared to Colombians, making their marginal health gains from system access potentially larger (Cubides et al., 2022, Black et al., 2015, Reed and Barbosa, 2017).

Second, migration could induce congestion in the host country’s health services, which, if present, might lead to overestimation of effects. However, international evidence, including the Syrian refugee crisis in Turkey, suggests such congestion effects are unlikely (Aygün et al., 2021). Third, the absence of detailed health data on Venezuelan migrants limits causal inference and precise health economic analyses. Availability of microdata before and after enrollment would allow robust impact evaluations. Finally, there is uncertainty about migrants’ access to healthcare through alternative means, which could overestimate impacts (Giraldo et al., 2022). Nevertheless, existing studies indicate regularization improves access to social services and subsidized health care (Ibáñez et al., 2022), and that irregular migrants face higher costs due to poorer health prior to care (Prada et al., 2023), making overestimation less probable than underestimation.

The methods of this study do not investigate potential sources of improvement for care in the system. For this, field studies are needed to gain a deeper understanding of barriers and opportunities. In particular, a better understanding of the role of investment in primary healthcare (PHC) is desirable. PHC offers efficiency in the financial sustainability of the health system (by reducing future costs), improves screening and early detection indicators for various diseases, reduces social inequities, and provides positive economic benefits by improving access to health services, among other factors (Bitton et al., 2017, Stange et al., 2023, Rowe et al., 2018). In fact, access to PHC has certainly generated substantial gains, making our underestimation of total benefits even more plausible.

## 5 Conclusion

The overarching goal of health systems is to achieve UHC. Under the SDGs, this implies reducing inequities in access and out-of-pocket expenses. Current evidence suggests that regularization of migrants in Colombia largely achieves this goal, and our study shows that the benefits from doing so are high. This holds true even under our fundamental assumption that rates the gains at a marginal value of the increase in spending (the CET) that is most likely an underestimate of the real impact of full access to the GSSHS. In this sense, Colombia's effort to adapt and adjust its health system in line with the UHC (Rivillas-García et al., 2021) has most likely been socially rewarded.

## Funding

Inter-American Development Bank.

## CRediT authorship contribution statement

**Oscar Espinosa:** Writing – review & editing, Writing – original draft, Visualization, Validation, Supervision, Software, Resources, Project administration, Methodology, Investigation, Formal analysis, Data curation, Conceptualization. **Paul Rodríguez:** Writing – review & editing, Writing – original draft, Visualization, Validation, Supervision, Software, Resources, Project administration, Methodology, Investigation, Formal analysis, Data curation, Conceptualization. **Valeria Bejarano:** Writing – review & editing, Writing – original draft, Visualization, Validation, Software, Formal analysis, Data curation. **Santiago León:** Writing – review & editing, Writing – original draft, Resources, Project administration. **José Luis Ortiz:** Writing – review & editing, Writing – original draft, Validation, Supervision, Resources, Project administration.

## Declaration of competing interest

The authors declare that they have no known competing financial interests or personal relationships that could have appeared to influence the work reported in this paper.

## Appendix Supplementary materials

Image, application 1

Image, application 2

Image, application 3

## Footnotes

1 Standardized health measure of disease burden that combines patient survival and health-related quality of life into a single index (Drummond et al., 2015, Turner et al., 2013).

2 A standardized health measure that seeks to measure the number of expected years of human life lost following the occurrence of an event, intervention, action or health course (Marshall, 2010).

3 For conversion to monthly terms, the annual value provided by the government is divided by twelve.

4 In accordance with the guidelines of the IETS and the iDSI Reference Case, a discount rate of 3% is assumed for both health outcomes and costs. It is also proposed to perform a sensitivity analysis at 0%, 6%, 9% and 12% (Ministerio de Salud y Protección Social 2022).

5 The dataset used for this development empirical considered expenditures in health services, health outcomes and other variables aggregated by HPS, region, disease group and year, for one lustrum (2013-2017) and for more than 10 million patients. Also, instrumental variable methods were used to solve the endogeneity problem between health spending and health outcomes.

6 With a confidence interval (CI) between 21.9% and 150.1% of GDPpc, as reported by Espinosa et al. (Espinosa et al., 2022).

7 With a CI between 19.8% and 129.3% of GDPpc, as reported by Espinosa et al. (Espinosa et al., 2022).

8 It is considered a CET as a percentage of GDPpc and not is as a deflated value (which takes 2019 as the base), given the advice of the IETS (Instituto de Evaluación Tecnológica en Salud, 2025), in addition to several consultations personal by the authors of this research with Professors Jessica Ochalek, Michael Drummond and Paul Revill of the *Center for Health Economics* at *the University of York.*

9 Given this context, only the application of the proposed methodology to female in the age groups 15-18, 19-44 and 45-49 were considered.

10 1 GDPpc and not 0.86 GDPpc, the point estimator of the CET, as a reference, is used since, by construction, using the CET point for the derivation of QALYs gained with the intervention, the IRR would be 0%.

11 The values are transformed to Colombian pesos using the average exchange rate for the year, the Colombian deflator is applied to bring it to 2021 prices and then translated back to USD. For example. The 1.228 million USD is equivalent to 3,370.44 million COP of 2015 (exchange rate of 2,744.66 USD/COP), which in 2021 prices corresponds to 4,435.56 million COP (approximately 31.6% cumulative inflation). The latter value, with a rate of 3,744.29 USD/COP, is equivalent to 1.185 million USD in 2021.

12 In 2021 it dropped to 73, due to COVID-19. To avoid 'inflating the calculation', we proceed to use 2019 as the reference year for life expectancy.
