# Supplementary material for: Health for all? A cost-utility evaluation of Colombia's policy to enroll Venezuelan migrants (2021–2023)
Source: J Migr Health. 2025 Oct 31;12:100374. doi: 10.1016/j.jmh.2025.100374 (PMC12661300; doi:10.1016/j.jmh.2025.100374)
Supplement: Supplementary file 2 [file mmc2.docx]

**Supplement 2. Venezuelans deaths and usage of the health system in Colombia**

In terms of mortality, the Venezuelan population presented 3,788 deaths between 2012 and 2022. Figure S1 presents the causes of death grouped according to the OECD classification (Table S1). Among them, HIV/AIDS and tuberculosis were the most frequent causes of death among men, particularly in 2018 and 2019, although these numbers have declined over the past three years. In 2019 there was a considerable increase in mortality related to pregnancy, births and the puerperium, as well as certain conditions originating in the perinatal period. However, these decreased in 2020, likely due to the COVID-19 pandemic. The impact of COVID-19 on mortality was notable in 2021, followed by a considerable reduction in 2022. Deaths were higher among men for causes such as accidents and assaults while cancer and diabetes showed a slight increase among women beginning in 2020. These causes, along with ischemic heart disease, have had the greatest impact on the migrant population.

**Table S1.** ICD-10 codes for causes of mortality according to the OECD

| **Cause of mortality** | **ICD-10 code** |
| --- | --- |
| Tuberculosis | A15-A19, B90 |
| HIV-AIDS | B20-B24 |
| Cancer (malignant neoplasms) | C00-C97 |
| Blood diseases | D50-D89 |
| Diabetes mellitus | E10-E14 |
| Parkinson's disease | G20-G21 |
| Alzheimer's disease | G30 |
| Hypertension | I10, I12, I15 |
| Ischaemic heart disease | I20-I25 |
| Cerebrovascular disease | I60-I69 |
| Influenza and pneumonia | J10-J18 |
| Chronic obstructive pulmonary disease | J40-J44 |
| Asthma | J45-J46 |
| Chronic liver disease and cirrhosis | K70, K73-K74 |
| Pregnancy, childbirth and the puerperium | O00-O99 |
| Certain conditions arising in the perinatal period | P00-P96 |
| Accidents | V01-X59, Y85-Y86 |
| Suicide (intentional self-harm) | X60-X84, Y87.0 |
| Assault | X85-Y09, Y87.1 |
| COVID-19 | U07.1, U07.2, U09.9, U10.9 |
| Social conflict | Z73.5 |

**Figure S1.** Number of deaths by sex and cause of mortality of Venezuelan migrants in Colombia, 2012-2022


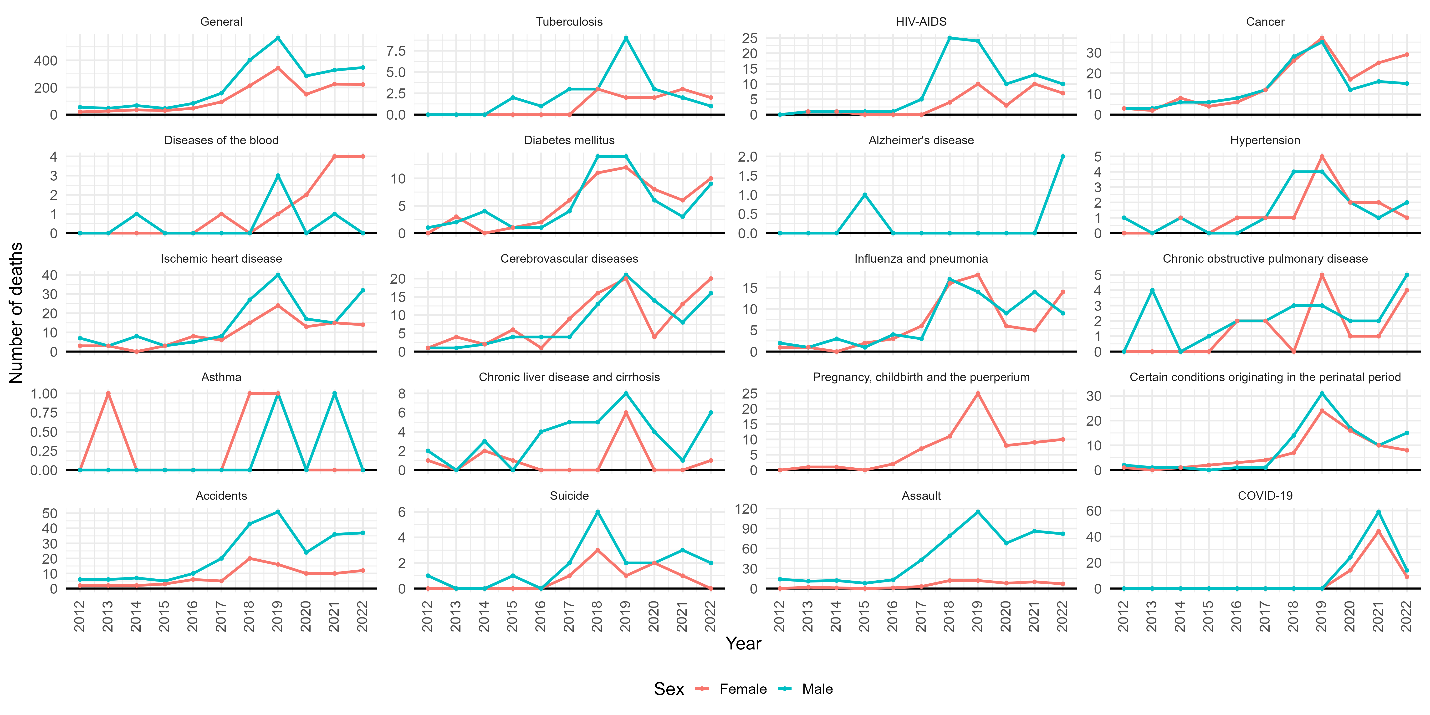


Figure S2 shows the number of healthcare services categorized according to the same health conditions mentioned above. Despite multiple limitations of the database of the Individual Health Services Provision Records (RIPS) by the MHSP -among them quality issues and underreporting-it can be observed that the most demanded health conditions among Venezuelan migrants enrolled to the subsidized scheme were related to pregnancy, births and puerperium, showing a notable increase between 2021 and 2023. They are followed, in order of frequency, by hypertension, HIV-AIDS, cancer and diabetes, with COVID-19 reaching similar values in 2021, a situation discussed in the works of Bojorquez et al. (2024) and Bowser et al. (2022). Other commonly treated conditions include blood diseases, asthma, influenza, ischemic heart disease and cerebrovascular disease.

**Figure S2.** Number of health care services use by sex and health conditions of Venezuelan migrants in Colombia, 2015-2023


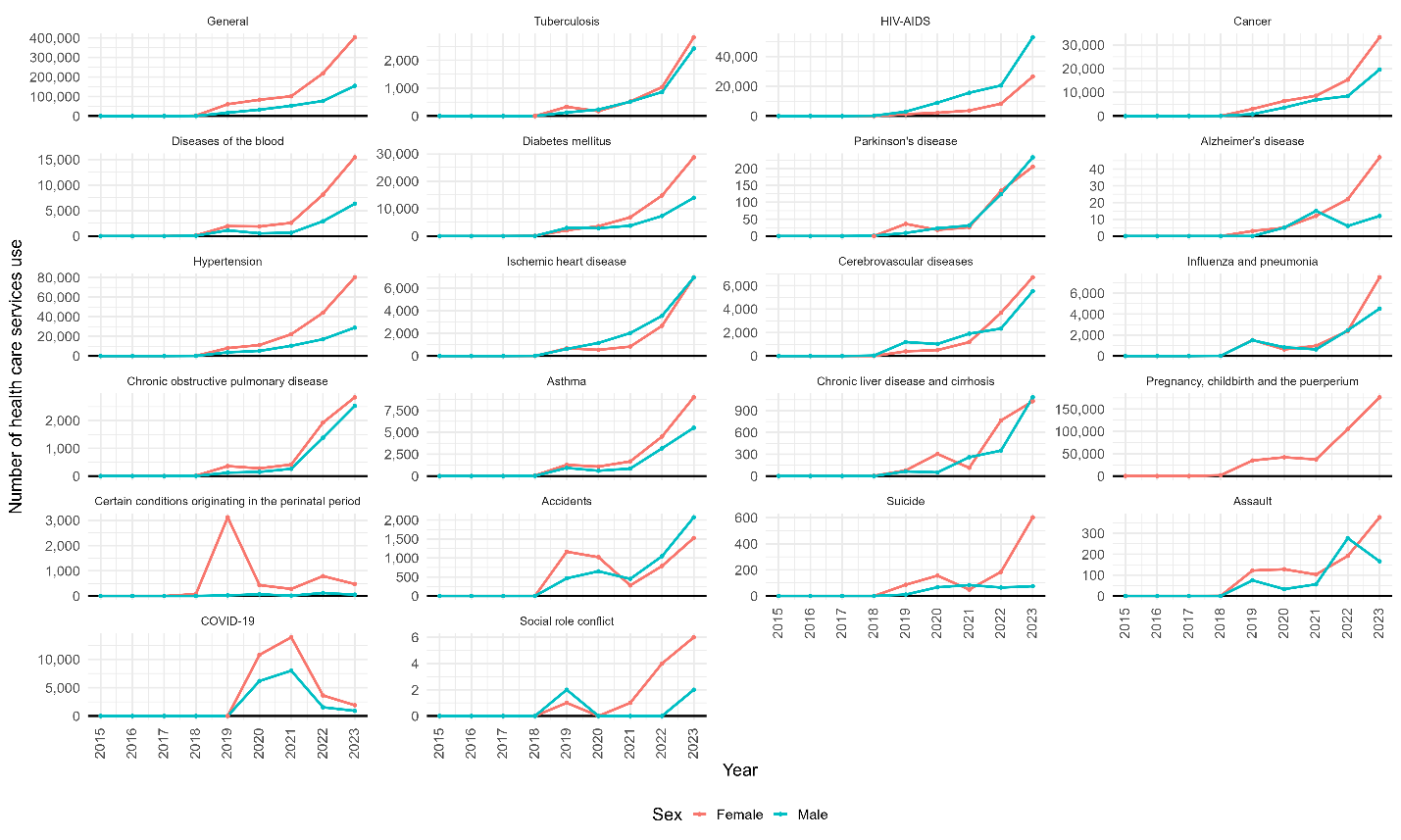


**References**

Bojorquez, I., Cubillos-Novella, A., Arroyo-Laguna, J., Martinez-Juarez, L., Sedas, A. C., Franco-Suarez, O., Suárez-Morales, Z., Adame-Avilés, E., Barragán-León, M., Suarez, A., Orcutt, M., & Spiegel, P. (2024). The response of health systems to the needs of migrants and refugees in the COVID-19 pandemic: a comparative case study between Mexico, Colombia and Peru. *The Lancet Regional Health - Americas*, 100763. https://doi.org/10.1016/j.lana.2024.100763

Bowser, D., Agarwal-Harding, P., Sombrio, A., Shepard, D., & Harker Roa, A. (2022). Integrating Venezuelan migrants into the Colombian health system during COVID-19. *Health Systems & Reform*, *8*(1). https://doi.org/10.1080/23288604.2022.2079448
