# Supplementary material for: Health for all? A cost-utility evaluation of Colombia's policy to enroll Venezuelan migrants (2021–2023)
Source: J Migr Health. 2025 Oct 31;12:100374. doi: 10.1016/j.jmh.2025.100374 (PMC12661300; doi:10.1016/j.jmh.2025.100374)
Supplement: Supplementary file 3 [file mmc3.docx]

**Supplement 3**

**Table S2.** Estimation of the generation of health outcomes by municipality in Colombia due to the CO-L1248 Programme, period 2021-2023.

| **Departament** | **Municipality** | **QALY** | | | **YLL** | | |
| --- | --- | --- | --- | --- | --- | --- | --- |
|  |  | **Lower limit** | **Point estimate** | **Upper limit** | **Lower limit** | **Point estimate** | **Upper limit** |
| Antioquia | Medellín | 365.7937 | 638.4552 | 2,502.9956 | 424.8077 | 737.0087 | 2,778.2558 |
| Antioquia | Abejorral | 0.2466 | 0.4304 | 1.6874 | 0.2864 | 0.4969 | 1.8730 |
| Antioquia | Abriaquí | 0.0000 | 0.0000 | 0.0000 | 0.0000 | 0.0000 | 0.0000 |
| Antioquia | Alejandría | 0.0673 | 0.1175 | 0.4608 | 0.0782 | 0.1357 | 0.5114 |
| Antioquia | Amagá | 2.7384 | 4.7796 | 18.7377 | 3.1802 | 5.5173 | 20.7984 |
| Antioquia | Amalfi | 0.2604 | 0.4545 | 1.7818 | 0.3024 | 0.5247 | 1.9777 |
| Antioquia | Andes | 1.5859 | 2.7679 | 10.8514 | 1.8417 | 3.1952 | 12.0448 |
| Antioquia | Angelópolis | 0.2379 | 0.4152 | 1.6276 | 0.2762 | 0.4792 | 1.8065 |
| Antioquia | Angostura | 0.0000 | 0.0000 | 0.0000 | 0.0000 | 0.0000 | 0.0000 |
| Antioquia | Anorí | 0.2017 | 0.3520 | 1.3798 | 0.2342 | 0.4063 | 1.5316 |
| Antioquia | Santafé de Antioquia | 2.4871 | 4.3410 | 17.0183 | 2.8883 | 5.0110 | 18.8898 |
| Antioquia | Anzá | 0.0588 | 0.1027 | 0.4025 | 0.0683 | 0.1185 | 0.4468 |
| Antioquia | Apartadó | 18.8084 | 32.8281 | 128.6992 | 21.8428 | 37.8956 | 142.8526 |
| Antioquia | Arboletes | 1.1626 | 2.0292 | 7.9555 | 1.3502 | 2.3425 | 8.8303 |
| Antioquia | Argelia | 0.0733 | 0.1279 | 0.5015 | 0.0851 | 0.1477 | 0.5567 |
| Antioquia | Armenia | 0.0183 | 0.0320 | 0.1255 | 0.0213 | 0.0370 | 0.1393 |
| Antioquia | Barbosa | 5.5798 | 9.7390 | 38.1808 | 6.4800 | 11.2424 | 42.3797 |
| Antioquia | Belmira | 0.0464 | 0.0810 | 0.3174 | 0.0539 | 0.0935 | 0.3523 |
| Antioquia | Bello | 65.9311 | 115.0759 | 451.1427 | 76.5678 | 132.8393 | 500.7559 |
| Antioquia | Betania | 0.1407 | 0.2456 | 0.9628 | 0.1634 | 0.2835 | 1.0686 |
| Antioquia | Betulia | 0.1242 | 0.2167 | 0.8495 | 0.1442 | 0.2501 | 0.9430 |
| Antioquia | Ciudad Bolívar | 1.0923 | 1.9066 | 7.4745 | 1.2686 | 2.2009 | 8.2965 |
| Antioquia | Briceño | 0.0200 | 0.0350 | 0.1370 | 0.0233 | 0.0404 | 0.1521 |
| Antioquia | Buriticá | 0.1613 | 0.2815 | 1.1037 | 0.1873 | 0.3250 | 1.2251 |
| Antioquia | Cáceres | 0.1156 | 0.2018 | 0.7913 | 0.1343 | 0.2330 | 0.8783 |
| Antioquia | Caicedo | 0.2554 | 0.4457 | 1.7475 | 0.2966 | 0.5145 | 1.9396 |
| Antioquia | Caldas | 6.5797 | 11.4843 | 45.0229 | 7.6413 | 13.2570 | 49.9741 |
| Antioquia | Campamento | 0.0072 | 0.0126 | 0.0492 | 0.0084 | 0.0145 | 0.0547 |
| Antioquia | Cañasgordas | 0.4619 | 0.8062 | 3.1608 | 0.5365 | 0.9307 | 3.5084 |
| Antioquia | Caracolí | 0.0656 | 0.1145 | 0.4487 | 0.0762 | 0.1321 | 0.4981 |
| Antioquia | Caramanta | 0.0312 | 0.0544 | 0.2133 | 0.0362 | 0.0628 | 0.2367 |
| Antioquia | Carepa | 4.9320 | 8.6083 | 33.7479 | 5.7277 | 9.9371 | 37.4593 |
| Antioquia | El Carmen de Viboral | 8.2542 | 14.4068 | 56.4804 | 9.5858 | 16.6307 | 62.6917 |
| Antioquia | Carolina | 0.0890 | 0.1553 | 0.6090 | 0.1034 | 0.1793 | 0.6759 |
| Antioquia | Caucasia | 6.6705 | 11.6427 | 45.6439 | 7.7467 | 13.4399 | 50.6635 |
| Antioquia | Chigorodó | 3.4008 | 5.9358 | 23.2706 | 3.9495 | 6.8521 | 25.8298 |
| Antioquia | Cisneros | 0.6602 | 1.1524 | 4.5178 | 0.7668 | 1.3303 | 5.0146 |
| Antioquia | Cocorná | 1.1747 | 2.0503 | 8.0380 | 1.3642 | 2.3668 | 8.9220 |
| Antioquia | Concepción | 0.0183 | 0.0319 | 0.1249 | 0.0212 | 0.0368 | 0.1386 |
| Antioquia | Concordia | 0.3753 | 0.6551 | 2.5684 | 0.4359 | 0.7563 | 2.8508 |
| Antioquia | Copacabana | 5.5295 | 9.6512 | 37.8363 | 6.4216 | 11.1409 | 41.9973 |
| Antioquia | Dabeiba | 0.4220 | 0.7365 | 2.8874 | 0.4900 | 0.8502 | 3.2049 |
| Antioquia | Don Matías | 2.0613 | 3.5978 | 14.1048 | 2.3939 | 4.1532 | 15.6559 |
| Antioquia | Ebéjico | 0.1168 | 0.2038 | 0.7989 | 0.1356 | 0.2352 | 0.8868 |
| Antioquia | El Bagre | 0.8824 | 1.5401 | 6.0379 | 1.0248 | 1.7779 | 6.7019 |
| Antioquia | Entrerrios | 0.5157 | 0.9001 | 3.5286 | 0.5989 | 1.0390 | 3.9167 |
| Antioquia | Envigado | 9.3692 | 16.3530 | 64.1101 | 10.8807 | 18.8773 | 71.1604 |
| Antioquia | Fredonia | 0.3758 | 0.6560 | 2.5717 | 0.4365 | 0.7572 | 2.8545 |
| Antioquia | Frontino | 0.2659 | 0.4641 | 1.8196 | 0.3088 | 0.5358 | 2.0197 |
| Antioquia | Giraldo | 0.2396 | 0.4182 | 1.6397 | 0.2783 | 0.4828 | 1.8200 |
| Antioquia | Girardota | 6.7904 | 11.8520 | 46.4645 | 7.8859 | 13.6815 | 51.5744 |
| Antioquia | Gómez Plata | 0.1364 | 0.2381 | 0.9335 | 0.1584 | 0.2749 | 1.0361 |
| Antioquia | Granada | 0.5420 | 0.9460 | 3.7088 | 0.6295 | 1.0921 | 4.1167 |
| Antioquia | Guadalupe | 0.0000 | 0.0000 | 0.0000 | 0.0000 | 0.0000 | 0.0000 |
| Antioquia | Guarne | 5.3924 | 9.4118 | 36.8981 | 6.2623 | 10.8647 | 40.9559 |
| Antioquia | Guatapé | 4.1373 | 7.2213 | 28.3104 | 4.8048 | 8.3360 | 31.4237 |
| Antioquia | Heliconia | 0.0750 | 0.1310 | 0.5134 | 0.0871 | 0.1512 | 0.5699 |
| Antioquia | Hispania | 0.0000 | 0.0000 | 0.0000 | 0.0000 | 0.0000 | 0.0000 |
| Antioquia | Itagüí | 36.5547 | 63.8025 | 250.1310 | 42.4522 | 73.6512 | 277.6385 |
| Antioquia | Ituango | 0.0496 | 0.0867 | 0.3397 | 0.0577 | 0.1000 | 0.3771 |
| Antioquia | Jardín | 0.3261 | 0.5692 | 2.2313 | 0.3787 | 0.6570 | 2.4767 |
| Antioquia | Jericó | 0.2759 | 0.4815 | 1.8878 | 0.3204 | 0.5559 | 2.0954 |
| Antioquia | La Ceja | 5.9927 | 10.4596 | 41.0056 | 6.9595 | 12.0741 | 45.5151 |
| Antioquia | La Estrella | 5.4938 | 9.5888 | 37.5919 | 6.3801 | 11.0690 | 41.7260 |
| Antioquia | La Pintada | 0.4735 | 0.8264 | 3.2399 | 0.5499 | 0.9540 | 3.5962 |
| Antioquia | La Unión | 1.2722 | 2.2206 | 8.7055 | 1.4775 | 2.5633 | 9.6629 |
| Antioquia | Liborina | 0.0468 | 0.0817 | 0.3201 | 0.0543 | 0.0943 | 0.3553 |
| Antioquia | Maceo | 0.3027 | 0.5283 | 2.0712 | 0.3515 | 0.6099 | 2.2989 |
| Antioquia | Marinilla | 14.7148 | 25.6832 | 100.6881 | 17.0888 | 29.6477 | 111.7610 |
| Antioquia | Montebello | 0.0193 | 0.0337 | 0.1322 | 0.0224 | 0.0389 | 0.1468 |
| Antioquia | Murindó | 0.0000 | 0.0000 | 0.0000 | 0.0000 | 0.0000 | 0.0000 |
| Antioquia | Mutatá | 1.1603 | 2.0252 | 7.9395 | 1.3475 | 2.3378 | 8.8126 |
| Antioquia | Nariño | 0.0385 | 0.0672 | 0.2633 | 0.0447 | 0.0775 | 0.2922 |
| Antioquia | Necoclí | 1.5762 | 2.7511 | 10.7854 | 1.8305 | 3.1758 | 11.9715 |
| Antioquia | Nechí | 0.1003 | 0.1751 | 0.6863 | 0.1165 | 0.2021 | 0.7618 |
| Antioquia | Olaya | 0.1167 | 0.2038 | 0.7988 | 0.1356 | 0.2352 | 0.8866 |
| Antioquia | Peñol | 3.9191 | 6.8404 | 26.8170 | 4.5514 | 7.8963 | 29.7661 |
| Antioquia | Peque | 0.0470 | 0.0820 | 0.3216 | 0.0546 | 0.0947 | 0.3570 |
| Antioquia | Pueblorrico | 0.0173 | 0.0302 | 0.1182 | 0.0201 | 0.0348 | 0.1312 |
| Antioquia | Puerto Berrío | 2.8419 | 4.9603 | 19.4464 | 3.3004 | 5.7260 | 21.5849 |
| Antioquia | Puerto Nare | 0.0701 | 0.1223 | 0.4794 | 0.0814 | 0.1412 | 0.5321 |
| Antioquia | Puerto Triunfo | 2.8216 | 4.9247 | 19.3069 | 3.2768 | 5.6849 | 21.4302 |
| Antioquia | Remedios | 1.6916 | 2.9525 | 11.5752 | 1.9645 | 3.4083 | 12.8481 |
| Antioquia | Retiro | 1.5709 | 2.7419 | 10.7493 | 1.8244 | 3.1651 | 11.9314 |
| Antioquia | Rionegro | 19.1700 | 33.4592 | 131.1734 | 22.2627 | 38.6241 | 145.5988 |
| Antioquia | Sabanalarga | 0.0000 | 0.0000 | 0.0000 | 0.0000 | 0.0000 | 0.0000 |
| Antioquia | Sabaneta | 14.7502 | 25.7449 | 100.9301 | 17.1298 | 29.7189 | 112.0296 |
| Antioquia | Salgar | 0.6322 | 1.1035 | 4.3260 | 0.7342 | 1.2738 | 4.8017 |
| Antioquia | San Andrés de Cuerquía | 0.1942 | 0.3389 | 1.3288 | 0.2255 | 0.3913 | 1.4749 |
| Antioquia | San Carlos | 0.4586 | 0.8004 | 3.1379 | 0.5326 | 0.9239 | 3.4830 |
| Antioquia | San Francisco | 0.0000 | 0.0000 | 0.0000 | 0.0000 | 0.0000 | 0.0000 |
| Antioquia | San Jerónimo | 5.1670 | 9.0185 | 35.3562 | 6.0006 | 10.4107 | 39.2444 |
| Antioquia | San José de La Montaña | 0.0000 | 0.0000 | 0.0000 | 0.0000 | 0.0000 | 0.0000 |
| Antioquia | San Juan de Urabá | 0.6061 | 1.0579 | 4.1475 | 0.7039 | 1.2212 | 4.6036 |
| Antioquia | San Luis | 0.9441 | 1.6478 | 6.4600 | 1.0964 | 1.9021 | 7.1704 |
| Antioquia | San Pedro de Los Milagros | 2.7050 | 4.7213 | 18.5093 | 3.1414 | 5.4501 | 20.5449 |
| Antioquia | San Pedro de Urabá | 0.2959 | 0.5165 | 2.0250 | 0.3437 | 0.5963 | 2.2477 |
| Antioquia | San Rafael | 0.8266 | 1.4428 | 5.6562 | 0.9600 | 1.6655 | 6.2782 |
| Antioquia | San Roque | 0.9423 | 1.6447 | 6.4479 | 1.0943 | 1.8986 | 7.1570 |
| Antioquia | San Vicente | 0.8385 | 1.4636 | 5.7377 | 0.9738 | 1.6895 | 6.3687 |
| Antioquia | Santa Bárbara | 0.6708 | 1.1708 | 4.5900 | 0.7790 | 1.3515 | 5.0948 |
| Antioquia | Santa Rosa de Osos | 1.6665 | 2.9088 | 11.4036 | 1.9354 | 3.3578 | 12.6576 |
| Antioquia | Santo Domingo | 0.4861 | 0.8484 | 3.3262 | 0.5645 | 0.9794 | 3.6919 |
| Antioquia | El Santuario | 7.5634 | 13.2011 | 51.7535 | 8.7836 | 15.2389 | 57.4450 |
| Antioquia | Segovia | 2.8615 | 4.9944 | 19.5800 | 3.3231 | 5.7653 | 21.7332 |
| Antioquia | Sonsón | 2.4252 | 4.2330 | 16.5949 | 2.8165 | 4.8864 | 18.4199 |
| Antioquia | Sopetrán | 3.1727 | 5.5376 | 21.7097 | 3.6846 | 6.3924 | 24.0971 |
| Antioquia | Támesis | 0.2377 | 0.4149 | 1.6267 | 0.2761 | 0.4790 | 1.8056 |
| Antioquia | Tarazá | 0.2256 | 0.3938 | 1.5437 | 0.2620 | 0.4545 | 1.7134 |
| Antioquia | Tarso | 0.0734 | 0.1282 | 0.5026 | 0.0853 | 0.1480 | 0.5578 |
| Antioquia | Titiribí | 0.0340 | 0.0593 | 0.2326 | 0.0395 | 0.0685 | 0.2582 |
| Antioquia | Toledo | 0.0000 | 0.0000 | 0.0000 | 0.0000 | 0.0000 | 0.0000 |
| Antioquia | Turbo | 11.8132 | 20.6187 | 80.8334 | 13.7190 | 23.8014 | 89.7228 |
| Antioquia | Uramita | 0.0099 | 0.0173 | 0.0677 | 0.0115 | 0.0199 | 0.0751 |
| Antioquia | Urrao | 0.2269 | 0.3960 | 1.5524 | 0.2635 | 0.4571 | 1.7231 |
| Antioquia | Valdivia | 0.0119 | 0.0208 | 0.0816 | 0.0139 | 0.0240 | 0.0906 |
| Antioquia | Valparaíso | 0.0000 | 0.0000 | 0.0000 | 0.0000 | 0.0000 | 0.0000 |
| Antioquia | Vegachí | 0.7735 | 1.3500 | 5.2924 | 0.8982 | 1.5584 | 5.8745 |
| Antioquia | Venecia | 0.2223 | 0.3880 | 1.5213 | 0.2582 | 0.4479 | 1.6885 |
| Antioquia | Vigía del Fuerte | 0.0000 | 0.0000 | 0.0000 | 0.0000 | 0.0000 | 0.0000 |
| Antioquia | Yalí | 0.1620 | 0.2828 | 1.1087 | 0.1882 | 0.3264 | 1.2306 |
| Antioquia | Yarumal | 0.6580 | 1.1484 | 4.5023 | 0.7641 | 1.3257 | 4.9974 |
| Antioquia | Yolombó | 0.7486 | 1.3067 | 5.1227 | 0.8694 | 1.5084 | 5.6860 |
| Antioquia | Yondó | 0.7188 | 1.2546 | 4.9186 | 0.8348 | 1.4483 | 5.4595 |
| Antioquia | Zaragoza | 0.3679 | 0.6422 | 2.5176 | 0.4273 | 0.7413 | 2.7945 |
| Atlántico | Barranquilla | 339.8381 | 593.1524 | 2,325.3909 | 394.6647 | 684.7128 | 2,581.1194 |
| Atlántico | Baranoa | 6.8142 | 11.8935 | 46.6273 | 7.9136 | 13.7294 | 51.7550 |
| Atlántico | Campo de La Cruz | 4.4756 | 7.8117 | 30.6251 | 5.1977 | 9.0176 | 33.9930 |
| Atlántico | Candelaria | 1.2077 | 2.1080 | 8.2641 | 1.4026 | 2.4334 | 9.1729 |
| Atlántico | Galapa | 12.8249 | 22.3846 | 87.7564 | 14.8940 | 25.8399 | 97.4071 |
| Atlántico | Juan de Acosta | 3.1859 | 5.5606 | 21.7997 | 3.6998 | 6.4189 | 24.1970 |
| Atlántico | Luruaco | 1.6464 | 2.8735 | 11.2654 | 1.9120 | 3.3171 | 12.5043 |
| Atlántico | Malambo | 18.1346 | 31.6520 | 124.0884 | 21.0602 | 36.5379 | 137.7346 |
| Atlántico | Manatí | 1.3485 | 2.3538 | 9.2276 | 1.5661 | 2.7171 | 10.2424 |
| Atlántico | Palmar de Varela | 1.7210 | 3.0038 | 11.7761 | 1.9986 | 3.4675 | 13.0711 |
| Atlántico | Piojó | 0.3244 | 0.5662 | 2.2196 | 0.3767 | 0.6535 | 2.4636 |
| Atlántico | Polonuevo | 2.4964 | 4.3573 | 17.0823 | 2.8992 | 5.0299 | 18.9609 |
| Atlántico | Ponedera | 1.8595 | 3.2456 | 12.7239 | 2.1595 | 3.7465 | 14.1231 |
| Atlántico | Puerto Colombia | 10.2295 | 17.8546 | 69.9971 | 11.8799 | 20.6107 | 77.6949 |
| Atlántico | Repelón | 2.1626 | 3.7745 | 14.7976 | 2.5114 | 4.3572 | 16.4249 |
| Atlántico | Sabanagrande | 3.6064 | 6.2945 | 24.6770 | 4.1882 | 7.2662 | 27.3908 |
| Atlántico | Sabanalarga | 13.3420 | 23.2871 | 91.2947 | 15.4945 | 26.8818 | 101.3346 |
| Atlántico | Santa Lucía | 3.5715 | 6.2337 | 24.4386 | 4.1477 | 7.1960 | 27.1262 |
| Atlántico | Santo Tomás | 4.4723 | 7.8059 | 30.6023 | 5.1938 | 9.0109 | 33.9677 |
| Atlántico | Soledad | 157.8776 | 275.5592 | 1,080.3004 | 183.3483 | 318.0951 | 1,199.1035 |
| Atlántico | Suan | 2.6243 | 4.5804 | 17.9570 | 3.0477 | 5.2875 | 19.9318 |
| Atlántico | Tubará | 2.1792 | 3.8035 | 14.9113 | 2.5307 | 4.3906 | 16.5511 |
| Atlántico | Usiacurí | 0.1766 | 0.3082 | 1.2083 | 0.2051 | 0.3558 | 1.3412 |
| Bogotá | Bogotá | 902.0933 | 1,574.5109 | 6,172.7025 | 1,047.6292 | 1,817.5562 | 6,851.5287 |
| Bolívar | Cartagena | 304.3356 | 531.1864 | 2,082.4600 | 353.4345 | 613.1817 | 2,311.4728 |
| Bolívar | Achí | 0.2961 | 0.5168 | 2.0261 | 0.3439 | 0.5966 | 2.2489 |
| Bolívar | Altos del Rosario | 0.1700 | 0.2967 | 1.1633 | 0.1974 | 0.3425 | 1.2912 |
| Bolívar | Arenal | 0.4897 | 0.8547 | 3.3506 | 0.5687 | 0.9866 | 3.7191 |
| Bolívar | Arjona | 5.2948 | 9.2416 | 36.2305 | 6.1490 | 10.6681 | 40.2149 |
| Bolívar | Arroyohondo | 0.4540 | 0.7923 | 3.1063 | 0.5272 | 0.9147 | 3.4479 |
| Bolívar | Barranco de Loba | 0.2948 | 0.5146 | 2.0175 | 0.3424 | 0.5940 | 2.2393 |
| Bolívar | Calamar | 1.1762 | 2.0529 | 8.0483 | 1.3660 | 2.3698 | 8.9334 |
| Bolívar | Cantagallo | 0.5552 | 0.9691 | 3.7993 | 0.6448 | 1.1187 | 4.2171 |
| Bolívar | Cicuco | 0.3169 | 0.5531 | 2.1682 | 0.3680 | 0.6384 | 2.4066 |
| Bolívar | Córdoba | 0.1109 | 0.1936 | 0.7589 | 0.1288 | 0.2235 | 0.8424 |
| Bolívar | Clemencia | 1.6436 | 2.8687 | 11.2463 | 1.9087 | 3.3115 | 12.4831 |
| Bolívar | El Carmen de Bolívar | 2.9612 | 5.1685 | 20.2627 | 3.4390 | 5.9664 | 22.4911 |
| Bolívar | El Guamo | 0.0809 | 0.1413 | 0.5539 | 0.0940 | 0.1631 | 0.6148 |
| Bolívar | El Peñón | 0.1259 | 0.2197 | 0.8615 | 0.1462 | 0.2537 | 0.9562 |
| Bolívar | Hatillo de Loba | 0.7141 | 1.2464 | 4.8862 | 0.8293 | 1.4387 | 5.4235 |
| Bolívar | Magangué | 8.4988 | 14.8338 | 58.1543 | 9.8699 | 17.1236 | 64.5496 |
| Bolívar | Mahates | 1.9536 | 3.4098 | 13.3678 | 2.2688 | 3.9362 | 14.8379 |
| Bolívar | Margarita | 0.3429 | 0.5986 | 2.3466 | 0.3983 | 0.6910 | 2.6047 |
| Bolívar | María La Baja | 2.6663 | 4.6538 | 18.2447 | 3.0965 | 5.3722 | 20.2511 |
| Bolívar | Montecristo | 0.4387 | 0.7657 | 3.0019 | 0.5095 | 0.8839 | 3.3320 |
| Bolívar | Mompós | 2.3268 | 4.0612 | 15.9215 | 2.7022 | 4.6881 | 17.6724 |
| Bolívar | Morales | 0.9183 | 1.6029 | 6.2839 | 1.0665 | 1.8503 | 6.9749 |
| Bolívar | Norosí | 1.0969 | 1.9146 | 7.5060 | 1.2739 | 2.2101 | 8.3314 |
| Bolívar | Pinillos | 0.2469 | 0.4309 | 1.6892 | 0.2867 | 0.4974 | 1.8750 |
| Bolívar | Regidor | 0.4639 | 0.8097 | 3.1744 | 0.5388 | 0.9347 | 3.5235 |
| Bolívar | Río Viejo | 0.4177 | 0.7290 | 2.8581 | 0.4851 | 0.8416 | 3.1724 |
| Bolívar | San Cristóbal | 0.6663 | 1.1629 | 4.5591 | 0.7738 | 1.3424 | 5.0605 |
| Bolívar | San Estanislao | 1.3851 | 2.4175 | 9.4775 | 1.6085 | 2.7907 | 10.5198 |
| Bolívar | San Fernando | 0.2464 | 0.4300 | 1.6859 | 0.2861 | 0.4964 | 1.8713 |
| Bolívar | San Jacinto | 0.7042 | 1.2291 | 4.8186 | 0.8178 | 1.4188 | 5.3485 |
| Bolívar | San Jacinto del Cauca | 0.0000 | 0.0000 | 0.0000 | 0.0000 | 0.0000 | 0.0000 |
| Bolívar | San Juan Nepomuceno | 2.8646 | 4.9998 | 19.6013 | 3.3267 | 5.7716 | 21.7569 |
| Bolívar | San Martín de Loba | 0.9604 | 1.6762 | 6.5714 | 1.1153 | 1.9350 | 7.2941 |
| Bolívar | San Pablo | 1.5292 | 2.6690 | 10.4636 | 1.7759 | 3.0810 | 11.6143 |
| Bolívar | Santa Catalina | 0.8860 | 1.5464 | 6.0625 | 1.0289 | 1.7851 | 6.7292 |
| Bolívar | Santa Rosa | 1.2022 | 2.0983 | 8.2262 | 1.3961 | 2.4222 | 9.1308 |
| Bolívar | Santa Rosa del Sur | 5.9935 | 10.4611 | 41.0117 | 6.9605 | 12.0759 | 45.5219 |
| Bolívar | Simití | 0.5613 | 0.9797 | 3.8408 | 0.6519 | 1.1309 | 4.2632 |
| Bolívar | Soplaviento | 0.2861 | 0.4993 | 1.9574 | 0.3322 | 0.5764 | 2.1726 |
| Bolívar | Talaigua Nuevo | 1.5144 | 2.6433 | 10.3626 | 1.7587 | 3.0513 | 11.5022 |
| Bolívar | Tiquisio | 0.2766 | 0.4828 | 1.8927 | 0.3212 | 0.5573 | 2.1009 |
| Bolívar | Turbaco | 8.7165 | 15.2137 | 59.6439 | 10.1227 | 17.5622 | 66.2030 |
| Bolívar | Turbaná | 0.2835 | 0.4949 | 1.9402 | 0.3293 | 0.5713 | 2.1536 |
| Bolívar | Villanueva | 0.7839 | 1.3683 | 5.3642 | 0.9104 | 1.5795 | 5.9541 |
| Bolívar | Zambrano | 0.1985 | 0.3465 | 1.3583 | 0.2305 | 0.4000 | 1.5077 |
| Boyacá | Tunja | 13.0298 | 22.7422 | 89.1582 | 15.1319 | 26.2527 | 98.9631 |
| Boyacá | Aquitania | 1.7467 | 3.0487 | 11.9520 | 2.0285 | 3.5193 | 13.2664 |
| Boyacá | Arcabuco | 0.3280 | 0.5725 | 2.2443 | 0.3809 | 0.6608 | 2.4911 |
| Boyacá | Belén | 0.4832 | 0.8435 | 3.3067 | 0.5612 | 0.9737 | 3.6703 |
| Boyacá | Boavita | 0.1517 | 0.2648 | 1.0380 | 0.1762 | 0.3057 | 1.1522 |
| Boyacá | Boyacá | 0.2136 | 0.3729 | 1.4618 | 0.2481 | 0.4304 | 1.6225 |
| Boyacá | Briceño | 0.0000 | 0.0000 | 0.0000 | 0.0000 | 0.0000 | 0.0000 |
| Boyacá | Buenavista | 0.0197 | 0.0345 | 0.1351 | 0.0229 | 0.0398 | 0.1499 |
| Boyacá | Busbanzá | 0.0000 | 0.0000 | 0.0000 | 0.0000 | 0.0000 | 0.0000 |
| Boyacá | Caldas | 0.0000 | 0.0000 | 0.0000 | 0.0000 | 0.0000 | 0.0000 |
| Boyacá | Campohermoso | 0.0021 | 0.0036 | 0.0142 | 0.0024 | 0.0042 | 0.0157 |
| Boyacá | Cerinza | 0.0380 | 0.0663 | 0.2600 | 0.0441 | 0.0765 | 0.2885 |
| Boyacá | Chinavita | 0.0000 | 0.0000 | 0.0000 | 0.0000 | 0.0000 | 0.0000 |
| Boyacá | Chiquinquirá | 2.0537 | 3.5844 | 14.0524 | 2.3850 | 4.1377 | 15.5978 |
| Boyacá | Chiscas | 0.0000 | 0.0000 | 0.0000 | 0.0000 | 0.0000 | 0.0000 |
| Boyacá | Chita | 0.4094 | 0.7146 | 2.8017 | 0.4755 | 0.8249 | 3.1098 |
| Boyacá | Chitaraque | 1.5012 | 2.6201 | 10.2719 | 1.7433 | 3.0246 | 11.4015 |
| Boyacá | Chivatá | 0.0000 | 0.0000 | 0.0000 | 0.0000 | 0.0000 | 0.0000 |
| Boyacá | Ciénega | 0.0088 | 0.0154 | 0.0605 | 0.0103 | 0.0178 | 0.0672 |
| Boyacá | Cómbita | 0.0387 | 0.0675 | 0.2646 | 0.0449 | 0.0779 | 0.2937 |
| Boyacá | Coper | 0.0000 | 0.0000 | 0.0000 | 0.0000 | 0.0000 | 0.0000 |
| Boyacá | Corrales | 0.1028 | 0.1795 | 0.7037 | 0.1194 | 0.2072 | 0.7811 |
| Boyacá | Covarachía | 0.1096 | 0.1912 | 0.7497 | 0.1272 | 0.2207 | 0.8321 |
| Boyacá | Cubará | 1.0906 | 1.9036 | 7.4627 | 1.2666 | 2.1974 | 8.2834 |
| Boyacá | Cucaita | 0.1810 | 0.3160 | 1.2387 | 0.2102 | 0.3647 | 1.3749 |
| Boyacá | Cuítiva | 0.0000 | 0.0000 | 0.0000 | 0.0000 | 0.0000 | 0.0000 |
| Boyacá | Chíquiza | 0.0000 | 0.0000 | 0.0000 | 0.0000 | 0.0000 | 0.0000 |
| Boyacá | Chivor | 0.0084 | 0.0146 | 0.0573 | 0.0097 | 0.0169 | 0.0636 |
| Boyacá | Duitama | 9.0458 | 15.7885 | 61.8972 | 10.5052 | 18.2257 | 68.7042 |
| Boyacá | El Cocuy | 0.0000 | 0.0000 | 0.0000 | 0.0000 | 0.0000 | 0.0000 |
| Boyacá | El Espino | 0.0389 | 0.0679 | 0.2661 | 0.0452 | 0.0783 | 0.2953 |
| Boyacá | Firavitoba | 0.1238 | 0.2161 | 0.8471 | 0.1438 | 0.2494 | 0.9403 |
| Boyacá | Floresta | 0.1295 | 0.2260 | 0.8860 | 0.1504 | 0.2609 | 0.9834 |
| Boyacá | Gachantivá | 0.1129 | 0.1971 | 0.7726 | 0.1311 | 0.2275 | 0.8576 |
| Boyacá | Gámeza | 0.1220 | 0.2129 | 0.8345 | 0.1416 | 0.2457 | 0.9263 |
| Boyacá | Garagoa | 0.3779 | 0.6595 | 2.5855 | 0.4388 | 0.7613 | 2.8698 |
| Boyacá | Guacamayas | 0.0113 | 0.0198 | 0.0776 | 0.0132 | 0.0229 | 0.0862 |
| Boyacá | Guateque | 0.4223 | 0.7371 | 2.8897 | 0.4904 | 0.8509 | 3.2075 |
| Boyacá | Guayatá | 0.0504 | 0.0879 | 0.3448 | 0.0585 | 0.1015 | 0.3827 |
| Boyacá | Güicán | 0.0021 | 0.0036 | 0.0142 | 0.0024 | 0.0042 | 0.0157 |
| Boyacá | Iza | 0.0057 | 0.0099 | 0.0389 | 0.0066 | 0.0115 | 0.0432 |
| Boyacá | Jenesano | 0.1956 | 0.3413 | 1.3381 | 0.2271 | 0.3940 | 1.4852 |
| Boyacá | Jericó | 0.1151 | 0.2010 | 0.7879 | 0.1337 | 0.2320 | 0.8746 |
| Boyacá | Labranzagrande | 0.0000 | 0.0000 | 0.0000 | 0.0000 | 0.0000 | 0.0000 |
| Boyacá | La Capilla | 0.0000 | 0.0000 | 0.0000 | 0.0000 | 0.0000 | 0.0000 |
| Boyacá | La Uvita | 0.0110 | 0.0192 | 0.0755 | 0.0128 | 0.0222 | 0.0838 |
| Boyacá | Villa de Leyva | 3.2087 | 5.6005 | 21.9561 | 3.7264 | 6.4650 | 24.3707 |
| Boyacá | Macanal | 0.0677 | 0.1182 | 0.4635 | 0.0787 | 0.1365 | 0.5145 |
| Boyacá | Maripí | 0.0000 | 0.0000 | 0.0000 | 0.0000 | 0.0000 | 0.0000 |
| Boyacá | Miraflores | 0.5643 | 0.9849 | 3.8612 | 0.6553 | 1.1369 | 4.2858 |
| Boyacá | Mongua | 0.1052 | 0.1835 | 0.7196 | 0.1221 | 0.2119 | 0.7987 |
| Boyacá | Monguí | 0.0412 | 0.0720 | 0.2821 | 0.0479 | 0.0831 | 0.3131 |
| Boyacá | Moniquirá | 4.3200 | 7.5402 | 29.5605 | 5.0170 | 8.7041 | 32.8113 |
| Boyacá | Motavita | 0.0086 | 0.0150 | 0.0588 | 0.0100 | 0.0173 | 0.0653 |
| Boyacá | Muzo | 0.2351 | 0.4104 | 1.6088 | 0.2730 | 0.4737 | 1.7857 |
| Boyacá | Nobsa | 1.1146 | 1.9454 | 7.6266 | 1.2944 | 2.2457 | 8.4653 |
| Boyacá | Nuevo Colón | 0.0534 | 0.0933 | 0.3657 | 0.0621 | 0.1077 | 0.4059 |
| Boyacá | Oicatá | 0.0573 | 0.1000 | 0.3921 | 0.0666 | 0.1155 | 0.4353 |
| Boyacá | Otanche | 0.0813 | 0.1420 | 0.5566 | 0.0945 | 0.1639 | 0.6178 |
| Boyacá | Pachavita | 0.0000 | 0.0000 | 0.0000 | 0.0000 | 0.0000 | 0.0000 |
| Boyacá | Páez | 0.0113 | 0.0198 | 0.0776 | 0.0132 | 0.0229 | 0.0862 |
| Boyacá | Paipa | 2.6611 | 4.6447 | 18.2089 | 3.0904 | 5.3616 | 20.2114 |
| Boyacá | Pajarito | 0.1062 | 0.1854 | 0.7269 | 0.1234 | 0.2140 | 0.8069 |
| Boyacá | Panqueba | 0.0928 | 0.1619 | 0.6348 | 0.1077 | 0.1869 | 0.7046 |
| Boyacá | Pauna | 0.0620 | 0.1082 | 0.4241 | 0.0720 | 0.1249 | 0.4707 |
| Boyacá | Paya | 0.0000 | 0.0000 | 0.0000 | 0.0000 | 0.0000 | 0.0000 |
| Boyacá | Paz de Río | 0.2068 | 0.3610 | 1.4152 | 0.2402 | 0.4167 | 1.5708 |
| Boyacá | Pesca | 0.0264 | 0.0461 | 0.1809 | 0.0307 | 0.0533 | 0.2007 |
| Boyacá | Pisba | 0.0000 | 0.0000 | 0.0000 | 0.0000 | 0.0000 | 0.0000 |
| Boyacá | Puerto Boyacá | 2.0964 | 3.6591 | 14.3452 | 2.4347 | 4.2239 | 15.9228 |
| Boyacá | Quípama | 0.0241 | 0.0420 | 0.1648 | 0.0280 | 0.0485 | 0.1830 |
| Boyacá | Ramiriquí | 0.2412 | 0.4210 | 1.6504 | 0.2801 | 0.4859 | 1.8319 |
| Boyacá | Ráquira | 2.1919 | 3.8257 | 14.9983 | 2.5455 | 4.4163 | 16.6477 |
| Boyacá | Rondón | 0.0000 | 0.0000 | 0.0000 | 0.0000 | 0.0000 | 0.0000 |
| Boyacá | Saboyá | 0.0217 | 0.0378 | 0.1484 | 0.0252 | 0.0437 | 0.1647 |
| Boyacá | Sáchica | 0.6972 | 1.2169 | 4.7707 | 0.8097 | 1.4047 | 5.2953 |
| Boyacá | Samacá | 2.2111 | 3.8593 | 15.1299 | 2.5678 | 4.4550 | 16.7938 |
| Boyacá | San José de Pare | 0.6978 | 1.2179 | 4.7745 | 0.8103 | 1.4059 | 5.2996 |
| Boyacá | San Luis de Gaceno | 0.4240 | 0.7400 | 2.9012 | 0.4924 | 0.8543 | 3.2203 |
| Boyacá | San Mateo | 0.0433 | 0.0755 | 0.2960 | 0.0502 | 0.0872 | 0.3286 |
| Boyacá | San Miguel de Sema | 0.0012 | 0.0020 | 0.0080 | 0.0014 | 0.0024 | 0.0089 |
| Boyacá | San Pablo de Borbur | 0.0980 | 0.1711 | 0.6708 | 0.1138 | 0.1975 | 0.7445 |
| Boyacá | Santana | 1.2544 | 2.1893 | 8.5831 | 1.4567 | 2.5273 | 9.5270 |
| Boyacá | Santa María | 0.1167 | 0.2037 | 0.7984 | 0.1355 | 0.2351 | 0.8862 |
| Boyacá | Santa Rosa de Viterbo | 0.5520 | 0.9635 | 3.7773 | 0.6411 | 1.1122 | 4.1927 |
| Boyacá | Santa Sofía | 0.3193 | 0.5573 | 2.1849 | 0.3708 | 0.6433 | 2.4251 |
| Boyacá | Sativanorte | 0.0000 | 0.0000 | 0.0000 | 0.0000 | 0.0000 | 0.0000 |
| Boyacá | Sativasur | 0.0000 | 0.0000 | 0.0000 | 0.0000 | 0.0000 | 0.0000 |
| Boyacá | Siachoque | 0.0000 | 0.0000 | 0.0000 | 0.0000 | 0.0000 | 0.0000 |
| Boyacá | Soatá | 0.4989 | 0.8707 | 3.4136 | 0.5793 | 1.0051 | 3.7890 |
| Boyacá | Socotá | 0.1762 | 0.3076 | 1.2059 | 0.2047 | 0.3551 | 1.3385 |
| Boyacá | Socha | 0.6458 | 1.1272 | 4.4191 | 0.7500 | 1.3012 | 4.9051 |
| Boyacá | Sogamoso | 9.4435 | 16.4827 | 64.6187 | 10.9671 | 19.0270 | 71.7249 |
| Boyacá | Somondoco | 0.0102 | 0.0178 | 0.0696 | 0.0118 | 0.0205 | 0.0773 |
| Boyacá | Sora | 0.0309 | 0.0539 | 0.2114 | 0.0359 | 0.0622 | 0.2346 |
| Boyacá | Sotaquirá | 0.0285 | 0.0498 | 0.1953 | 0.0331 | 0.0575 | 0.2167 |
| Boyacá | Soracá | 0.0723 | 0.1263 | 0.4950 | 0.0840 | 0.1458 | 0.5494 |
| Boyacá | Susacón | 0.0000 | 0.0000 | 0.0000 | 0.0000 | 0.0000 | 0.0000 |
| Boyacá | Sutamarchán | 1.0357 | 1.8077 | 7.0871 | 1.2028 | 2.0868 | 7.8664 |
| Boyacá | Sutatenza | 0.0000 | 0.0000 | 0.0000 | 0.0000 | 0.0000 | 0.0000 |
| Boyacá | Tasco | 0.0478 | 0.0834 | 0.3271 | 0.0555 | 0.0963 | 0.3631 |
| Boyacá | Tenza | 0.0000 | 0.0000 | 0.0000 | 0.0000 | 0.0000 | 0.0000 |
| Boyacá | Tibaná | 0.0840 | 0.1466 | 0.5747 | 0.0975 | 0.1692 | 0.6379 |
| Boyacá | Tibasosa | 0.9796 | 1.7098 | 6.7030 | 1.1376 | 1.9737 | 7.4401 |
| Boyacá | Tinjacá | 0.2632 | 0.4594 | 1.8011 | 0.3057 | 0.5303 | 1.9991 |
| Boyacá | Tipacoque | 0.1289 | 0.2249 | 0.8819 | 0.1497 | 0.2597 | 0.9789 |
| Boyacá | Toca | 0.6760 | 1.1799 | 4.6257 | 0.7851 | 1.3620 | 5.1344 |
| Boyacá | Togüí | 0.1715 | 0.2993 | 1.1735 | 0.1992 | 0.3455 | 1.3026 |
| Boyacá | Tópaga | 0.1189 | 0.2076 | 0.8139 | 0.1381 | 0.2397 | 0.9034 |
| Boyacá | Tota | 0.0324 | 0.0566 | 0.2220 | 0.0377 | 0.0654 | 0.2464 |
| Boyacá | Tununguá | 0.0000 | 0.0000 | 0.0000 | 0.0000 | 0.0000 | 0.0000 |
| Boyacá | Turmequé | 0.1021 | 0.1781 | 0.6983 | 0.1185 | 0.2056 | 0.7751 |
| Boyacá | Tuta | 0.4328 | 0.7555 | 2.9618 | 0.5027 | 0.8721 | 3.2875 |
| Boyacá | Tutazá | 0.0147 | 0.0257 | 0.1008 | 0.0171 | 0.0297 | 0.1119 |
| Boyacá | Umbita | 0.0294 | 0.0514 | 0.2014 | 0.0342 | 0.0593 | 0.2235 |
| Boyacá | Ventaquemada | 0.7757 | 1.3538 | 5.3076 | 0.9008 | 1.5628 | 5.8913 |
| Boyacá | Viracachá | 0.0000 | 0.0000 | 0.0000 | 0.0000 | 0.0000 | 0.0000 |
| Boyacá | Zetaquira | 0.0020 | 0.0035 | 0.0135 | 0.0023 | 0.0040 | 0.0150 |
| Caldas | Manizales | 24.5063 | 42.7731 | 167.6875 | 28.4599 | 49.3757 | 186.1285 |
| Caldas | Aguadas | 0.5179 | 0.9039 | 3.5436 | 0.6014 | 1.0434 | 3.9332 |
| Caldas | Anserma | 0.9029 | 1.5760 | 6.1785 | 1.0486 | 1.8193 | 6.8580 |
| Caldas | Aranzazu | 0.7281 | 1.2709 | 4.9823 | 0.8456 | 1.4671 | 5.5303 |
| Caldas | Belalcázar | 0.1883 | 0.3287 | 1.2887 | 0.2187 | 0.3795 | 1.4304 |
| Caldas | Chinchiná | 2.5375 | 4.4290 | 17.3635 | 2.9469 | 5.1127 | 19.2730 |
| Caldas | Filadelfia | 0.1051 | 0.1835 | 0.7194 | 0.1221 | 0.2118 | 0.7985 |
| Caldas | La Dorada | 0.9004 | 1.5716 | 6.1611 | 1.0457 | 1.8141 | 6.8386 |
| Caldas | La Merced | 0.1001 | 0.1748 | 0.6852 | 0.1163 | 0.2018 | 0.7605 |
| Caldas | Manzanares | 0.2761 | 0.4820 | 1.8894 | 0.3207 | 0.5563 | 2.0972 |
| Caldas | Marmato | 1.3976 | 2.4393 | 9.5631 | 1.6230 | 2.8159 | 10.6147 |
| Caldas | Marquetalia | 0.1126 | 0.1966 | 0.7708 | 0.1308 | 0.2270 | 0.8555 |
| Caldas | Marulanda | 0.0000 | 0.0000 | 0.0000 | 0.0000 | 0.0000 | 0.0000 |
| Caldas | Neira | 0.9874 | 1.7234 | 6.7564 | 1.1467 | 1.9894 | 7.4995 |
| Caldas | Norcasia | 0.0644 | 0.1123 | 0.4404 | 0.0747 | 0.1297 | 0.4889 |
| Caldas | Pácora | 0.5102 | 0.8905 | 3.4910 | 0.5925 | 1.0279 | 3.8749 |
| Caldas | Palestina | 0.8327 | 1.4535 | 5.6982 | 0.9671 | 1.6778 | 6.3248 |
| Caldas | Pensilvania | 0.0344 | 0.0600 | 0.2354 | 0.0399 | 0.0693 | 0.2612 |
| Caldas | Riosucio | 1.0798 | 1.8846 | 7.3884 | 1.2539 | 2.1755 | 8.2009 |
| Caldas | Risaralda | 0.3569 | 0.6230 | 2.4423 | 0.4145 | 0.7191 | 2.7109 |
| Caldas | Salamina | 0.2167 | 0.3783 | 1.4830 | 0.2517 | 0.4367 | 1.6461 |
| Caldas | Samaná | 0.1199 | 0.2094 | 0.8207 | 0.1393 | 0.2417 | 0.9110 |
| Caldas | San José | 0.0728 | 0.1271 | 0.4983 | 0.0846 | 0.1467 | 0.5531 |
| Caldas | Supía | 1.0890 | 1.9007 | 7.4514 | 1.2646 | 2.1941 | 8.2708 |
| Caldas | Victoria | 0.0275 | 0.0479 | 0.1879 | 0.0319 | 0.0553 | 0.2085 |
| Caldas | Villamaría | 1.1350 | 1.9810 | 7.7662 | 1.3181 | 2.2868 | 8.6203 |
| Caldas | Viterbo | 0.3212 | 0.5607 | 2.1981 | 0.3731 | 0.6472 | 2.4399 |
| Caquetá | Florencia | 2.2351 | 3.9011 | 15.2940 | 2.5957 | 4.5033 | 16.9759 |
| Caquetá | Albania | 0.0000 | 0.0000 | 0.0000 | 0.0000 | 0.0000 | 0.0000 |
| Caquetá | Belén de Los Andaquíes | 0.0000 | 0.0000 | 0.0000 | 0.0000 | 0.0000 | 0.0000 |
| Caquetá | Cartagena del Chairá | 0.0590 | 0.1031 | 0.4041 | 0.0686 | 0.1190 | 0.4485 |
| Caquetá | Curillo | 0.0130 | 0.0226 | 0.0887 | 0.0151 | 0.0261 | 0.0984 |
| Caquetá | El Doncello | 0.0266 | 0.0465 | 0.1823 | 0.0309 | 0.0537 | 0.2024 |
| Caquetá | El Paujil | 0.0000 | 0.0000 | 0.0000 | 0.0000 | 0.0000 | 0.0000 |
| Caquetá | La Montañita | 0.0000 | 0.0000 | 0.0000 | 0.0000 | 0.0000 | 0.0000 |
| Caquetá | Milán | 0.0000 | 0.0000 | 0.0000 | 0.0000 | 0.0000 | 0.0000 |
| Caquetá | Morelia | 0.0000 | 0.0000 | 0.0000 | 0.0000 | 0.0000 | 0.0000 |
| Caquetá | Puerto Rico | 0.0545 | 0.0951 | 0.3727 | 0.0633 | 0.1097 | 0.4137 |
| Caquetá | San José del Fragua | 0.0566 | 0.0988 | 0.3872 | 0.0657 | 0.1140 | 0.4298 |
| Caquetá | San Vicente del Caguán | 0.4452 | 0.7770 | 3.0462 | 0.5170 | 0.8969 | 3.3812 |
| Caquetá | Solita | 0.0000 | 0.0000 | 0.0000 | 0.0000 | 0.0000 | 0.0000 |
| Caquetá | Valparaíso | 0.0000 | 0.0000 | 0.0000 | 0.0000 | 0.0000 | 0.0000 |
| Cauca | Popayán | 19.6639 | 34.3212 | 134.5527 | 22.8363 | 39.6191 | 149.3498 |
| Cauca | Almaguer | 0.0000 | 0.0000 | 0.0000 | 0.0000 | 0.0000 | 0.0000 |
| Cauca | Argelia | 1.0427 | 1.8199 | 7.1347 | 1.2109 | 2.1008 | 7.9194 |
| Cauca | Balboa | 0.1499 | 0.2616 | 1.0256 | 0.1741 | 0.3020 | 1.1384 |
| Cauca | Bolívar | 0.2165 | 0.3779 | 1.4815 | 0.2514 | 0.4362 | 1.6445 |
| Cauca | Buenos Aires | 0.5648 | 0.9857 | 3.8645 | 0.6559 | 1.1379 | 4.2895 |
| Cauca | Cajibío | 0.0787 | 0.1373 | 0.5383 | 0.0914 | 0.1585 | 0.5975 |
| Cauca | Caldono | 0.1344 | 0.2345 | 0.9195 | 0.1560 | 0.2707 | 1.0206 |
| Cauca | Caloto | 0.3841 | 0.6704 | 2.6284 | 0.4461 | 0.7739 | 2.9174 |
| Cauca | Corinto | 0.8833 | 1.5417 | 6.0442 | 1.0258 | 1.7797 | 6.7089 |
| Cauca | El Tambo | 0.1689 | 0.2948 | 1.1559 | 0.1962 | 0.3403 | 1.2830 |
| Cauca | Florencia | 0.0088 | 0.0154 | 0.0604 | 0.0102 | 0.0178 | 0.0670 |
| Cauca | Guachené | 0.5285 | 0.9225 | 3.6165 | 0.6138 | 1.0649 | 4.0142 |
| Cauca | Guapi | 0.0568 | 0.0992 | 0.3887 | 0.0660 | 0.1145 | 0.4315 |
| Cauca | Inzá | 0.1554 | 0.2712 | 1.0631 | 0.1804 | 0.3130 | 1.1800 |
| Cauca | La Sierra | 0.0676 | 0.1180 | 0.4627 | 0.0785 | 0.1362 | 0.5136 |
| Cauca | La Vega | 0.0000 | 0.0000 | 0.0000 | 0.0000 | 0.0000 | 0.0000 |
| Cauca | López | 0.0130 | 0.0226 | 0.0887 | 0.0151 | 0.0261 | 0.0984 |
| Cauca | Mercaderes | 0.4354 | 0.7600 | 2.9796 | 0.5057 | 0.8773 | 3.3073 |
| Cauca | Miranda | 2.1063 | 3.6763 | 14.4125 | 2.4461 | 4.2438 | 15.9974 |
| Cauca | Morales | 0.4028 | 0.7031 | 2.7564 | 0.4678 | 0.8116 | 3.0595 |
| Cauca | Padilla | 0.3034 | 0.5295 | 2.0759 | 0.3523 | 0.6112 | 2.3042 |
| Cauca | Paéz | 0.0543 | 0.0948 | 0.3715 | 0.0631 | 0.1094 | 0.4124 |
| Cauca | Patía | 3.2246 | 5.6283 | 22.0651 | 3.7449 | 6.4971 | 24.4916 |
| Cauca | Piamonte | 0.0000 | 0.0000 | 0.0000 | 0.0000 | 0.0000 | 0.0000 |
| Cauca | Piendamó | 1.8360 | 3.2045 | 12.5630 | 2.1322 | 3.6992 | 13.9446 |
| Cauca | Puerto Tejada | 4.8463 | 8.4587 | 33.1616 | 5.6282 | 9.7645 | 36.8085 |
| Cauca | Puracé | 0.0000 | 0.0000 | 0.0000 | 0.0000 | 0.0000 | 0.0000 |
| Cauca | Rosas | 0.1157 | 0.2020 | 0.7918 | 0.1344 | 0.2331 | 0.8789 |
| Cauca | San Sebastián | 0.0000 | 0.0000 | 0.0000 | 0.0000 | 0.0000 | 0.0000 |
| Cauca | Santander de Quilichao | 11.5493 | 20.1580 | 79.0275 | 13.4125 | 23.2697 | 87.7183 |
| Cauca | Santa Rosa | 0.0094 | 0.0164 | 0.0643 | 0.0109 | 0.0189 | 0.0714 |
| Cauca | Silvia | 0.0486 | 0.0848 | 0.3324 | 0.0564 | 0.0979 | 0.3690 |
| Cauca | Sotará | 0.0000 | 0.0000 | 0.0000 | 0.0000 | 0.0000 | 0.0000 |
| Cauca | Suárez | 1.1653 | 2.0339 | 7.9737 | 1.3533 | 2.3478 | 8.8505 |
| Cauca | Sucre | 0.0066 | 0.0115 | 0.0453 | 0.0077 | 0.0133 | 0.0503 |
| Cauca | Timbío | 0.8197 | 1.4308 | 5.6092 | 0.9520 | 1.6516 | 6.2260 |
| Cauca | Timbiquí | 0.0110 | 0.0192 | 0.0755 | 0.0128 | 0.0222 | 0.0838 |
| Cauca | Toribío | 0.0799 | 0.1394 | 0.5465 | 0.0928 | 0.1609 | 0.6066 |
| Cauca | Totoró | 0.0603 | 0.1053 | 0.4129 | 0.0701 | 0.1216 | 0.4583 |
| Cauca | Villa Rica | 2.4538 | 4.2828 | 16.7903 | 2.8497 | 4.9439 | 18.6368 |
| Cesar | Valledupar | 142.5124 | 248.7406 | 975.1611 | 165.5041 | 287.1368 | 1,082.4018 |
| Cesar | Aguachica | 16.9382 | 29.5639 | 115.9022 | 19.6709 | 34.1275 | 128.6482 |
| Cesar | Agustín Codazzi | 10.2019 | 17.8064 | 69.8080 | 11.8478 | 20.5550 | 77.4849 |
| Cesar | Astrea | 1.3891 | 2.4245 | 9.5050 | 1.6132 | 2.7988 | 10.5503 |
| Cesar | Becerril | 3.3196 | 5.7940 | 22.7147 | 3.8551 | 6.6884 | 25.2127 |
| Cesar | Bosconia | 2.8707 | 5.0104 | 19.6429 | 3.3338 | 5.7839 | 21.8030 |
| Cesar | Chimichagua | 1.1971 | 2.0894 | 8.1915 | 1.3903 | 2.4120 | 9.0923 |
| Cesar | Chiriguaná | 2.7561 | 4.8104 | 18.8588 | 3.2007 | 5.5530 | 20.9328 |
| Cesar | Curumaní | 8.3255 | 14.5313 | 56.9685 | 9.6687 | 16.7744 | 63.2335 |
| Cesar | El Copey | 1.2376 | 2.1602 | 8.4688 | 1.4373 | 2.4936 | 9.4001 |
| Cesar | El Paso | 5.7097 | 9.9657 | 39.0693 | 6.6308 | 11.5040 | 43.3658 |
| Cesar | Gamarra | 0.7196 | 1.2561 | 4.9243 | 0.8357 | 1.4499 | 5.4658 |
| Cesar | González | 0.1859 | 0.3245 | 1.2724 | 0.2159 | 0.3746 | 1.4123 |
| Cesar | La Gloria | 1.0233 | 1.7861 | 7.0021 | 1.1884 | 2.0618 | 7.7721 |
| Cesar | La Jagua de Ibirico | 5.7588 | 10.0514 | 39.4055 | 6.6879 | 11.6030 | 43.7390 |
| Cesar | Manaure | 2.2584 | 3.9417 | 15.4531 | 2.6227 | 4.5502 | 17.1525 |
| Cesar | Pailitas | 1.7618 | 3.0750 | 12.0550 | 2.0460 | 3.5496 | 13.3808 |
| Cesar | Pelaya | 1.3791 | 2.4070 | 9.4366 | 1.6016 | 2.7786 | 10.4743 |
| Cesar | Pueblo Bello | 1.0062 | 1.7562 | 6.8850 | 1.1685 | 2.0273 | 7.6421 |
| Cesar | Río de Oro | 0.3671 | 0.6407 | 2.5119 | 0.4263 | 0.7396 | 2.7882 |
| Cesar | La Paz | 4.4379 | 7.7459 | 30.3670 | 5.1539 | 8.9416 | 33.7065 |
| Cesar | San Alberto | 2.2029 | 3.8449 | 15.0734 | 2.5583 | 4.4384 | 16.7311 |
| Cesar | San Diego | 1.8176 | 3.1725 | 12.4374 | 2.1109 | 3.6622 | 13.8052 |
| Cesar | San Martín | 3.4954 | 6.1008 | 23.9176 | 4.0593 | 7.0426 | 26.5479 |
| Cesar | Tamalameque | 0.9349 | 1.6317 | 6.3969 | 1.0857 | 1.8836 | 7.1004 |
| Córdoba | Montería | 21.1344 | 36.8880 | 144.6153 | 24.5441 | 42.5821 | 160.5190 |
| Córdoba | Ayapel | 0.0986 | 0.1721 | 0.6748 | 0.1145 | 0.1987 | 0.7490 |
| Córdoba | Buenavista | 0.0613 | 0.1070 | 0.4193 | 0.0712 | 0.1235 | 0.4655 |
| Córdoba | Canalete | 0.0395 | 0.0689 | 0.2703 | 0.0459 | 0.0796 | 0.3000 |
| Córdoba | Cereté | 2.1355 | 3.7272 | 14.6122 | 2.4800 | 4.3026 | 16.2191 |
| Córdoba | Chimá | 0.1894 | 0.3305 | 1.2958 | 0.2199 | 0.3816 | 1.4383 |
| Córdoba | Chinú | 0.9776 | 1.7063 | 6.6895 | 1.1353 | 1.9697 | 7.4251 |
| Córdoba | Ciénaga de Oro | 1.3728 | 2.3961 | 9.3938 | 1.5943 | 2.7660 | 10.4269 |
| Córdoba | Cotorra | 0.2974 | 0.5191 | 2.0351 | 0.3454 | 0.5992 | 2.2589 |
| Córdoba | La Apartada | 0.0293 | 0.0512 | 0.2006 | 0.0340 | 0.0591 | 0.2226 |
| Córdoba | Lorica | 3.4538 | 6.0283 | 23.6333 | 4.0110 | 6.9588 | 26.2323 |
| Córdoba | Los Córdobas | 0.0525 | 0.0917 | 0.3594 | 0.0610 | 0.1058 | 0.3990 |
| Córdoba | Momil | 0.4175 | 0.7287 | 2.8566 | 0.4848 | 0.8411 | 3.1708 |
| Córdoba | Montelíbano | 1.2168 | 2.1239 | 8.3264 | 1.4132 | 2.4517 | 9.2421 |
| Córdoba | Moñitos | 0.5288 | 0.9230 | 3.6185 | 0.6141 | 1.0655 | 4.0165 |
| Córdoba | Planeta Rica | 0.5721 | 0.9986 | 3.9148 | 0.6644 | 1.1527 | 4.3454 |
| Córdoba | Pueblo Nuevo | 0.1760 | 0.3071 | 1.2040 | 0.2043 | 0.3545 | 1.3364 |
| Córdoba | Puerto Escondido | 0.0760 | 0.1327 | 0.5201 | 0.0883 | 0.1531 | 0.5773 |
| Córdoba | Puerto Libertador | 0.2479 | 0.4327 | 1.6962 | 0.2879 | 0.4995 | 1.8828 |
| Córdoba | Purísima | 0.3660 | 0.6388 | 2.5044 | 0.4251 | 0.7374 | 2.7799 |
| Córdoba | Sahagún | 1.9595 | 3.4201 | 13.4083 | 2.2756 | 3.9481 | 14.8828 |
| Córdoba | San Andrés Sotavento | 0.0786 | 0.1372 | 0.5377 | 0.0913 | 0.1583 | 0.5969 |
| Córdoba | San Antero | 0.4626 | 0.8074 | 3.1653 | 0.5372 | 0.9320 | 3.5134 |
| Córdoba | San Bernardo del Viento | 0.6773 | 1.1822 | 4.6346 | 0.7866 | 1.3647 | 5.1443 |
| Córdoba | San Carlos | 0.0829 | 0.1447 | 0.5673 | 0.0963 | 0.1671 | 0.6297 |
| Córdoba | San José de Ure | 0.0397 | 0.0693 | 0.2718 | 0.0461 | 0.0800 | 0.3017 |
| Córdoba | San Pelayo | 0.3147 | 0.5493 | 2.1536 | 0.3655 | 0.6341 | 2.3905 |
| Córdoba | Tierralta | 0.7114 | 1.2416 | 4.8677 | 0.8261 | 1.4333 | 5.4030 |
| Córdoba | Tuchín | 0.1001 | 0.1747 | 0.6850 | 0.1163 | 0.2017 | 0.7604 |
| Córdoba | Valencia | 0.3917 | 0.6837 | 2.6805 | 0.4549 | 0.7893 | 2.9752 |
| Cundinamarca | Agua de Dios | 0.1495 | 0.2609 | 1.0227 | 0.1736 | 0.3011 | 1.1352 |
| Cundinamarca | Albán | 0.2365 | 0.4128 | 1.6183 | 0.2747 | 0.4765 | 1.7963 |
| Cundinamarca | Anapoima | 0.9015 | 1.5735 | 6.1689 | 1.0470 | 1.8164 | 6.8473 |
| Cundinamarca | Anolaima | 0.5416 | 0.9454 | 3.7063 | 0.6290 | 1.0913 | 4.1139 |
| Cundinamarca | Arbeláez | 0.1965 | 0.3429 | 1.3445 | 0.2282 | 0.3959 | 1.4923 |
| Cundinamarca | Beltrán | 0.0000 | 0.0000 | 0.0000 | 0.0000 | 0.0000 | 0.0000 |
| Cundinamarca | Bituima | 0.0056 | 0.0097 | 0.0381 | 0.0065 | 0.0112 | 0.0423 |
| Cundinamarca | Bojacá | 0.2203 | 0.3846 | 1.5076 | 0.2559 | 0.4439 | 1.6734 |
| Cundinamarca | Cabrera | 0.0000 | 0.0000 | 0.0000 | 0.0000 | 0.0000 | 0.0000 |
| Cundinamarca | Cachipay | 0.2443 | 0.4264 | 1.6718 | 0.2837 | 0.4922 | 1.8556 |
| Cundinamarca | Cajicá | 5.6521 | 9.8652 | 38.6755 | 6.5640 | 11.3880 | 42.9288 |
| Cundinamarca | Caparrapí | 0.1218 | 0.2127 | 0.8338 | 0.1415 | 0.2455 | 0.9255 |
| Cundinamarca | Cáqueza | 1.1913 | 2.0794 | 8.1520 | 1.3835 | 2.4003 | 9.0484 |
| Cundinamarca | Carmen de Carupa | 0.1196 | 0.2088 | 0.8186 | 0.1389 | 0.2410 | 0.9086 |
| Cundinamarca | Chaguaní | 0.1480 | 0.2583 | 1.0127 | 0.1719 | 0.2982 | 1.1241 |
| Cundinamarca | Chía | 16.2965 | 28.4439 | 111.5112 | 18.9256 | 32.8345 | 123.7743 |
| Cundinamarca | Chipaque | 0.1846 | 0.3222 | 1.2633 | 0.2144 | 0.3720 | 1.4023 |
| Cundinamarca | Choachí | 0.4959 | 0.8656 | 3.3934 | 0.5759 | 0.9992 | 3.7666 |
| Cundinamarca | Chocontá | 1.6463 | 2.8735 | 11.2653 | 1.9119 | 3.3171 | 12.5042 |
| Cundinamarca | Cogua | 0.4242 | 0.7403 | 2.9024 | 0.4926 | 0.8546 | 3.2216 |
| Cundinamarca | Cota | 4.6862 | 8.1793 | 32.0662 | 5.4423 | 9.4419 | 35.5926 |
| Cundinamarca | Cucunubá | 0.1365 | 0.2383 | 0.9340 | 0.1585 | 0.2750 | 1.0368 |
| Cundinamarca | El Colegio | 1.3246 | 2.3119 | 9.0637 | 1.5383 | 2.6688 | 10.0604 |
| Cundinamarca | El Peñón | 0.0242 | 0.0422 | 0.1655 | 0.0281 | 0.0487 | 0.1837 |
| Cundinamarca | El Rosal | 1.9268 | 3.3630 | 13.1844 | 2.2377 | 3.8822 | 14.6344 |
| Cundinamarca | Facatativá | 10.6991 | 18.6742 | 73.2102 | 12.4252 | 21.5568 | 81.2613 |
| Cundinamarca | Fómeque | 1.3030 | 2.2742 | 8.9158 | 1.5132 | 2.6253 | 9.8963 |
| Cundinamarca | Fosca | 0.2379 | 0.4152 | 1.6276 | 0.2762 | 0.4792 | 1.8065 |
| Cundinamarca | Funza | 5.2018 | 9.0791 | 35.5937 | 6.0410 | 10.4806 | 39.5081 |
| Cundinamarca | Fúquene | 0.2121 | 0.3701 | 1.4510 | 0.2463 | 0.4273 | 1.6106 |
| Cundinamarca | Fusagasugá | 7.2471 | 12.6490 | 49.5892 | 8.4163 | 14.6016 | 55.0426 |
| Cundinamarca | Gachalá | 0.0000 | 0.0000 | 0.0000 | 0.0000 | 0.0000 | 0.0000 |
| Cundinamarca | Gachancipá | 0.7402 | 1.2919 | 5.0647 | 0.8596 | 1.4913 | 5.6217 |
| Cundinamarca | Gachetá | 0.1301 | 0.2271 | 0.8901 | 0.1511 | 0.2621 | 0.9880 |
| Cundinamarca | Gama | 0.0000 | 0.0000 | 0.0000 | 0.0000 | 0.0000 | 0.0000 |
| Cundinamarca | Girardot | 4.7867 | 8.3547 | 32.7535 | 5.5589 | 9.6443 | 36.3555 |
| Cundinamarca | Granada | 0.5303 | 0.9256 | 3.6287 | 0.6159 | 1.0685 | 4.0277 |
| Cundinamarca | Guachetá | 0.2642 | 0.4611 | 1.8076 | 0.3068 | 0.5323 | 2.0064 |
| Cundinamarca | Guaduas | 1.9537 | 3.4099 | 13.3683 | 2.2689 | 3.9363 | 14.8385 |
| Cundinamarca | Guasca | 0.5101 | 0.8903 | 3.4903 | 0.5924 | 1.0277 | 3.8741 |
| Cundinamarca | Guataquí | 0.0124 | 0.0217 | 0.0850 | 0.0144 | 0.0250 | 0.0943 |
| Cundinamarca | Guatavita | 0.1205 | 0.2104 | 0.8249 | 0.1400 | 0.2429 | 0.9156 |
| Cundinamarca | Guayabal de Síquima | 0.2994 | 0.5226 | 2.0487 | 0.3477 | 0.6032 | 2.2740 |
| Cundinamarca | Guayabetal | 0.2117 | 0.3695 | 1.4487 | 0.2459 | 0.4266 | 1.6080 |
| Cundinamarca | Gutiérrez | 0.3134 | 0.5470 | 2.1444 | 0.3639 | 0.6314 | 2.3802 |
| Cundinamarca | Jerusalén | 0.0039 | 0.0068 | 0.0268 | 0.0046 | 0.0079 | 0.0298 |
| Cundinamarca | Junín | 0.0000 | 0.0000 | 0.0000 | 0.0000 | 0.0000 | 0.0000 |
| Cundinamarca | La Calera | 1.4897 | 2.6001 | 10.1936 | 1.7301 | 3.0015 | 11.3146 |
| Cundinamarca | La Mesa | 3.1851 | 5.5593 | 21.7948 | 3.6990 | 6.4175 | 24.1916 |
| Cundinamarca | La Palma | 0.1689 | 0.2948 | 1.1556 | 0.1961 | 0.3403 | 1.2827 |
| Cundinamarca | La Peña | 0.0119 | 0.0207 | 0.0812 | 0.0138 | 0.0239 | 0.0902 |
| Cundinamarca | La Vega | 2.1570 | 3.7649 | 14.7599 | 2.5050 | 4.3461 | 16.3831 |
| Cundinamarca | Lenguazaque | 0.4135 | 0.7217 | 2.8292 | 0.4802 | 0.8331 | 3.1403 |
| Cundinamarca | Machetá | 0.1587 | 0.2770 | 1.0859 | 0.1843 | 0.3197 | 1.2053 |
| Cundinamarca | Madrid | 9.2226 | 16.0970 | 63.1067 | 10.7105 | 18.5818 | 70.0467 |
| Cundinamarca | Manta | 0.0038 | 0.0067 | 0.0262 | 0.0045 | 0.0077 | 0.0291 |
| Cundinamarca | Medina | 0.2845 | 0.4965 | 1.9465 | 0.3304 | 0.5732 | 2.1606 |
| Cundinamarca | Mosquera | 8.9782 | 15.6705 | 61.4344 | 10.4266 | 18.0894 | 68.1905 |
| Cundinamarca | Nariño | 0.0000 | 0.0000 | 0.0000 | 0.0000 | 0.0000 | 0.0000 |
| Cundinamarca | Nemocón | 0.3833 | 0.6690 | 2.6226 | 0.4451 | 0.7722 | 2.9110 |
| Cundinamarca | Nilo | 0.0574 | 0.1001 | 0.3925 | 0.0666 | 0.1156 | 0.4356 |
| Cundinamarca | Nimaima | 0.0818 | 0.1427 | 0.5595 | 0.0950 | 0.1647 | 0.6210 |
| Cundinamarca | Nocaima | 0.2275 | 0.3970 | 1.5564 | 0.2642 | 0.4583 | 1.7276 |
| Cundinamarca | Venecia | 0.0871 | 0.1521 | 0.5963 | 0.1012 | 0.1756 | 0.6619 |
| Cundinamarca | Pacho | 1.1457 | 1.9998 | 7.8399 | 1.3306 | 2.3084 | 8.7020 |
| Cundinamarca | Paime | 0.0161 | 0.0281 | 0.1102 | 0.0187 | 0.0325 | 0.1224 |
| Cundinamarca | Pandi | 0.1444 | 0.2520 | 0.9881 | 0.1677 | 0.2909 | 1.0967 |
| Cundinamarca | Paratebueno | 0.5008 | 0.8741 | 3.4267 | 0.5816 | 1.0090 | 3.8035 |
| Cundinamarca | Pasca | 0.1612 | 0.2814 | 1.1032 | 0.1872 | 0.3248 | 1.2246 |
| Cundinamarca | Puerto Salgar | 0.1879 | 0.3280 | 1.2860 | 0.2183 | 0.3787 | 1.4274 |
| Cundinamarca | Pulí | 0.0113 | 0.0198 | 0.0776 | 0.0132 | 0.0229 | 0.0862 |
| Cundinamarca | Quebradanegra | 0.2129 | 0.3715 | 1.4565 | 0.2472 | 0.4289 | 1.6167 |
| Cundinamarca | Quetame | 0.0493 | 0.0860 | 0.3370 | 0.0572 | 0.0992 | 0.3741 |
| Cundinamarca | Quipile | 0.0780 | 0.1361 | 0.5336 | 0.0906 | 0.1571 | 0.5923 |
| Cundinamarca | Apulo | 0.1678 | 0.2929 | 1.1483 | 0.1949 | 0.3381 | 1.2746 |
| Cundinamarca | Ricaurte | 0.4896 | 0.8545 | 3.3500 | 0.5686 | 0.9864 | 3.7184 |
| Cundinamarca | San Antonio del Tequendama | 0.3864 | 0.6745 | 2.6442 | 0.4488 | 0.7786 | 2.9350 |
| Cundinamarca | San Bernardo | 0.3812 | 0.6653 | 2.6084 | 0.4427 | 0.7680 | 2.8952 |
| Cundinamarca | San Cayetano | 0.0204 | 0.0355 | 0.1393 | 0.0236 | 0.0410 | 0.1546 |
| Cundinamarca | San Francisco | 1.1995 | 2.0936 | 8.2077 | 1.3930 | 2.4168 | 9.1104 |
| Cundinamarca | San Juan de Río Seco | 0.2449 | 0.4275 | 1.6759 | 0.2844 | 0.4935 | 1.8602 |
| Cundinamarca | Sasaima | 0.6942 | 1.2116 | 4.7500 | 0.8062 | 1.3986 | 5.2723 |
| Cundinamarca | Sesquilé | 0.4803 | 0.8384 | 3.2867 | 0.5578 | 0.9678 | 3.6482 |
| Cundinamarca | Sibaté | 2.6507 | 4.6265 | 18.1377 | 3.0783 | 5.3407 | 20.1324 |
| Cundinamarca | Silvania | 1.7456 | 3.0468 | 11.9447 | 2.0272 | 3.5171 | 13.2583 |
| Cundinamarca | Simijaca | 0.1367 | 0.2385 | 0.9352 | 0.1587 | 0.2754 | 1.0380 |
| Cundinamarca | Soacha | 96.7887 | 168.9346 | 662.2903 | 112.4037 | 195.0118 | 735.1239 |
| Cundinamarca | Sopó | 0.6825 | 1.1913 | 4.6702 | 0.7926 | 1.3751 | 5.1838 |
| Cundinamarca | Subachoque | 0.8447 | 1.4743 | 5.7798 | 0.9809 | 1.7019 | 6.4154 |
| Cundinamarca | Suesca | 1.3274 | 2.3169 | 9.0831 | 1.5416 | 2.6745 | 10.0820 |
| Cundinamarca | Supatá | 0.1322 | 0.2307 | 0.9044 | 0.1535 | 0.2663 | 1.0038 |
| Cundinamarca | Susa | 0.0488 | 0.0851 | 0.3338 | 0.0566 | 0.0983 | 0.3705 |
| Cundinamarca | Sutatausa | 0.1169 | 0.2041 | 0.8000 | 0.1358 | 0.2356 | 0.8879 |
| Cundinamarca | Tabio | 0.7212 | 1.2588 | 4.9352 | 0.8376 | 1.4532 | 5.4779 |
| Cundinamarca | Tausa | 0.2325 | 0.4058 | 1.5910 | 0.2700 | 0.4685 | 1.7660 |
| Cundinamarca | Tena | 0.2953 | 0.5154 | 2.0207 | 0.3430 | 0.5950 | 2.2429 |
| Cundinamarca | Tenjo | 0.8587 | 1.4988 | 5.8760 | 0.9973 | 1.7302 | 6.5222 |
| Cundinamarca | Tibacuy | 0.0201 | 0.0350 | 0.1373 | 0.0233 | 0.0404 | 0.1524 |
| Cundinamarca | Tibirita | 0.0000 | 0.0000 | 0.0000 | 0.0000 | 0.0000 | 0.0000 |
| Cundinamarca | Tocaima | 0.5976 | 1.0430 | 4.0889 | 0.6940 | 1.2040 | 4.5385 |
| Cundinamarca | Tocancipá | 1.9423 | 3.3902 | 13.2907 | 2.2557 | 3.9135 | 14.7524 |
| Cundinamarca | Topaipí | 0.0000 | 0.0000 | 0.0000 | 0.0000 | 0.0000 | 0.0000 |
| Cundinamarca | Ubalá | 0.0420 | 0.0732 | 0.2871 | 0.0487 | 0.0845 | 0.3186 |
| Cundinamarca | Ubaque | 0.1099 | 0.1918 | 0.7518 | 0.1276 | 0.2214 | 0.8345 |
| Cundinamarca | Villa de San Diego de Ubate | 3.0510 | 5.3252 | 20.8767 | 3.5432 | 6.1472 | 23.1726 |
| Cundinamarca | Une | 0.3106 | 0.5421 | 2.1251 | 0.3607 | 0.6257 | 2.3588 |
| Cundinamarca | Útica | 0.1083 | 0.1890 | 0.7411 | 0.1258 | 0.2182 | 0.8226 |
| Cundinamarca | Vergara | 0.0916 | 0.1599 | 0.6268 | 0.1064 | 0.1846 | 0.6957 |
| Cundinamarca | Vianí | 0.1597 | 0.2788 | 1.0931 | 0.1855 | 0.3219 | 1.2133 |
| Cundinamarca | Villagómez | 0.0554 | 0.0968 | 0.3794 | 0.0644 | 0.1117 | 0.4211 |
| Cundinamarca | Villapinzón | 0.3208 | 0.5598 | 2.1948 | 0.3725 | 0.6463 | 2.4362 |
| Cundinamarca | Villeta | 3.0367 | 5.3002 | 20.7790 | 3.5266 | 6.1184 | 23.0642 |
| Cundinamarca | Viotá | 0.1777 | 0.3102 | 1.2160 | 0.2064 | 0.3581 | 1.3498 |
| Cundinamarca | Yacopí | 0.0000 | 0.0000 | 0.0000 | 0.0000 | 0.0000 | 0.0000 |
| Cundinamarca | Zipacón | 0.1682 | 0.2936 | 1.1510 | 0.1954 | 0.3389 | 1.2776 |
| Cundinamarca | Zipaquirá | 10.5781 | 18.4630 | 72.3824 | 12.2847 | 21.3130 | 80.3424 |
| Chocó | Quibdó | 1.4032 | 2.4491 | 9.6015 | 1.6296 | 2.8272 | 10.6574 |
| Chocó | Acandí | 0.0867 | 0.1513 | 0.5932 | 0.1007 | 0.1747 | 0.6584 |
| Chocó | Alto Baudó | 0.0000 | 0.0000 | 0.0000 | 0.0000 | 0.0000 | 0.0000 |
| Chocó | Atrato | 0.0153 | 0.0266 | 0.1045 | 0.0177 | 0.0308 | 0.1160 |
| Chocó | Bagadó | 0.0000 | 0.0000 | 0.0000 | 0.0000 | 0.0000 | 0.0000 |
| Chocó | Bahía Solano | 0.0200 | 0.0350 | 0.1370 | 0.0233 | 0.0404 | 0.1521 |
| Chocó | Bajo Baudó | 0.0000 | 0.0000 | 0.0000 | 0.0000 | 0.0000 | 0.0000 |
| Chocó | Bojayá | 0.0000 | 0.0000 | 0.0000 | 0.0000 | 0.0000 | 0.0000 |
| Chocó | El Cantón del San Pablo | 0.0675 | 0.1179 | 0.4621 | 0.0784 | 0.1361 | 0.5130 |
| Chocó | Carmen del Darién | 0.0000 | 0.0000 | 0.0000 | 0.0000 | 0.0000 | 0.0000 |
| Chocó | Cértegui | 0.0000 | 0.0000 | 0.0000 | 0.0000 | 0.0000 | 0.0000 |
| Chocó | Condoto | 0.0000 | 0.0000 | 0.0000 | 0.0000 | 0.0000 | 0.0000 |
| Chocó | El Carmen de Atrato | 0.3953 | 0.6899 | 2.7048 | 0.4591 | 0.7964 | 3.0023 |
| Chocó | El Litoral del San Juan | 0.0000 | 0.0000 | 0.0000 | 0.0000 | 0.0000 | 0.0000 |
| Chocó | Istmina | 0.1683 | 0.2938 | 1.1517 | 0.1955 | 0.3391 | 1.2784 |
| Chocó | Juradó | 0.0180 | 0.0313 | 0.1229 | 0.0209 | 0.0362 | 0.1364 |
| Chocó | Medio Baudó | 0.0021 | 0.0036 | 0.0142 | 0.0024 | 0.0042 | 0.0157 |
| Chocó | Nóvita | 0.0000 | 0.0000 | 0.0000 | 0.0000 | 0.0000 | 0.0000 |
| Chocó | Nuquí | 0.0000 | 0.0000 | 0.0000 | 0.0000 | 0.0000 | 0.0000 |
| Chocó | Río Iró | 0.0000 | 0.0000 | 0.0000 | 0.0000 | 0.0000 | 0.0000 |
| Chocó | Río Quito | 0.0000 | 0.0000 | 0.0000 | 0.0000 | 0.0000 | 0.0000 |
| Chocó | Riosucio | 0.3826 | 0.6677 | 2.6177 | 0.4443 | 0.7708 | 2.9056 |
| Chocó | San José del Palmar | 0.0000 | 0.0000 | 0.0000 | 0.0000 | 0.0000 | 0.0000 |
| Chocó | Sipí | 0.0000 | 0.0000 | 0.0000 | 0.0000 | 0.0000 | 0.0000 |
| Chocó | Tadó | 0.0284 | 0.0496 | 0.1943 | 0.0330 | 0.0572 | 0.2157 |
| Chocó | Unguía | 0.1188 | 0.2074 | 0.8131 | 0.1380 | 0.2394 | 0.9025 |
| Chocó | Unión Panamericana | 0.0954 | 0.1665 | 0.6526 | 0.1108 | 0.1921 | 0.7243 |
| Huila | Neiva | 10.1722 | 17.7545 | 69.6046 | 11.8133 | 20.4951 | 77.2592 |
| Huila | Acevedo | 0.4638 | 0.8094 | 3.1733 | 0.5386 | 0.9344 | 3.5223 |
| Huila | Agrado | 0.0349 | 0.0609 | 0.2387 | 0.0405 | 0.0703 | 0.2650 |
| Huila | Aipe | 0.0730 | 0.1274 | 0.4994 | 0.0848 | 0.1471 | 0.5544 |
| Huila | Algeciras | 0.3521 | 0.6145 | 2.4092 | 0.4089 | 0.7094 | 2.6741 |
| Huila | Altamira | 0.0102 | 0.0178 | 0.0696 | 0.0118 | 0.0205 | 0.0773 |
| Huila | Baraya | 0.0487 | 0.0849 | 0.3329 | 0.0565 | 0.0980 | 0.3695 |
| Huila | Campoalegre | 0.3790 | 0.6615 | 2.5934 | 0.4401 | 0.7636 | 2.8786 |
| Huila | Colombia | 0.0030 | 0.0053 | 0.0207 | 0.0035 | 0.0061 | 0.0230 |
| Huila | Elías | 0.0643 | 0.1122 | 0.4401 | 0.0747 | 0.1296 | 0.4885 |
| Huila | Garzón | 1.2255 | 2.1390 | 8.3858 | 1.4232 | 2.4692 | 9.3080 |
| Huila | Gigante | 0.4811 | 0.8398 | 3.2922 | 0.5587 | 0.9694 | 3.6542 |
| Huila | Guadalupe | 0.5468 | 0.9543 | 3.7412 | 0.6350 | 1.1016 | 4.1527 |
| Huila | Hobo | 0.0829 | 0.1447 | 0.5674 | 0.0963 | 0.1671 | 0.6298 |
| Huila | Iquira | 0.0162 | 0.0282 | 0.1107 | 0.0188 | 0.0326 | 0.1228 |
| Huila | Isnos | 0.3482 | 0.6077 | 2.3826 | 0.4044 | 0.7016 | 2.6446 |
| Huila | La Argentina | 0.0711 | 0.1241 | 0.4865 | 0.0826 | 0.1433 | 0.5400 |
| Huila | La Plata | 0.6057 | 1.0572 | 4.1445 | 0.7034 | 1.2204 | 4.6003 |
| Huila | Nátaga | 0.0000 | 0.0000 | 0.0000 | 0.0000 | 0.0000 | 0.0000 |
| Huila | Oporapa | 0.0645 | 0.1125 | 0.4411 | 0.0749 | 0.1299 | 0.4896 |
| Huila | Paicol | 0.0762 | 0.1329 | 0.5211 | 0.0884 | 0.1534 | 0.5784 |
| Huila | Palermo | 0.1755 | 0.3064 | 1.2012 | 0.2039 | 0.3537 | 1.3333 |
| Huila | Palestina | 0.1563 | 0.2728 | 1.0695 | 0.1815 | 0.3149 | 1.1871 |
| Huila | Pital | 0.1647 | 0.2875 | 1.1273 | 0.1913 | 0.3319 | 1.2512 |
| Huila | Pitalito | 4.5067 | 7.8660 | 30.8377 | 5.2338 | 9.0802 | 34.2290 |
| Huila | Rivera | 0.4272 | 0.7456 | 2.9231 | 0.4961 | 0.8607 | 3.2445 |
| Huila | Saladoblanco | 0.0451 | 0.0787 | 0.3086 | 0.0524 | 0.0909 | 0.3425 |
| Huila | San Agustín | 0.1470 | 0.2565 | 1.0056 | 0.1707 | 0.2961 | 1.1161 |
| Huila | Santa María | 0.2005 | 0.3499 | 1.3716 | 0.2328 | 0.4039 | 1.5225 |
| Huila | Suaza | 0.2464 | 0.4300 | 1.6860 | 0.2861 | 0.4964 | 1.8714 |
| Huila | Tarqui | 0.0510 | 0.0890 | 0.3488 | 0.0592 | 0.1027 | 0.3872 |
| Huila | Tesalia | 0.1644 | 0.2869 | 1.1246 | 0.1909 | 0.3311 | 1.2483 |
| Huila | Tello | 0.1286 | 0.2245 | 0.8799 | 0.1493 | 0.2591 | 0.9767 |
| Huila | Teruel | 0.1060 | 0.1850 | 0.7254 | 0.1231 | 0.2136 | 0.8052 |
| Huila | Timaná | 0.3253 | 0.5677 | 2.2258 | 0.3778 | 0.6554 | 2.4706 |
| Huila | Villavieja | 0.0468 | 0.0817 | 0.3202 | 0.0543 | 0.0943 | 0.3554 |
| Huila | Yaguará | 0.0648 | 0.1132 | 0.4437 | 0.0753 | 0.1306 | 0.4925 |
| La Guajira | Riohacha | 156.6014 | 273.3317 | 1,071.5678 | 181.8662 | 315.5238 | 1,189.4105 |
| La Guajira | Albania | 6.5107 | 11.3638 | 44.5507 | 7.5611 | 13.1180 | 49.4500 |
| La Guajira | Barrancas | 9.1032 | 15.8886 | 62.2897 | 10.5718 | 18.3412 | 69.1398 |
| La Guajira | Dibulla | 9.3040 | 16.2392 | 63.6642 | 10.8051 | 18.7460 | 70.6655 |
| La Guajira | Distracción | 2.6183 | 4.5700 | 17.9161 | 3.0407 | 5.2754 | 19.8863 |
| La Guajira | El Molino | 2.1666 | 3.7815 | 14.8250 | 2.5161 | 4.3652 | 16.4553 |
| La Guajira | Fonseca | 25.5071 | 44.5201 | 174.5362 | 29.6222 | 51.3923 | 193.7304 |
| La Guajira | Hatonuevo | 6.6063 | 11.5306 | 45.2043 | 7.6721 | 13.3104 | 50.1756 |
| La Guajira | La Jagua del Pilar | 0.3153 | 0.5503 | 2.1573 | 0.3661 | 0.6352 | 2.3946 |
| La Guajira | Maicao | 157.6083 | 275.0890 | 1,078.4570 | 183.0354 | 317.5523 | 1,197.0574 |
| La Guajira | Manaure | 8.2491 | 14.3980 | 56.4459 | 9.5800 | 16.6205 | 62.6534 |
| La Guajira | San Juan del Cesar | 12.6264 | 22.0380 | 86.3978 | 14.6634 | 25.4399 | 95.8992 |
| La Guajira | Uribia | 29.9348 | 52.2481 | 204.8332 | 34.7642 | 60.3133 | 227.3592 |
| La Guajira | Urumita | 1.2928 | 2.2565 | 8.8462 | 1.5014 | 2.6048 | 9.8190 |
| La Guajira | Villanueva | 6.2631 | 10.9315 | 42.8559 | 7.2735 | 12.6189 | 47.5688 |
| Magdalena | Santa Marta | 169.1185 | 295.1789 | 1,157.2175 | 196.4026 | 340.7434 | 1,284.4793 |
| Magdalena | Algarrobo | 0.5349 | 0.9335 | 3.6598 | 0.6211 | 1.0776 | 4.0623 |
| Magdalena | Aracataca | 3.2062 | 5.5962 | 21.9392 | 3.7235 | 6.4600 | 24.3519 |
| Magdalena | Ariguaní | 0.5251 | 0.9165 | 3.5931 | 0.6098 | 1.0580 | 3.9882 |
| Magdalena | Cerro San Antonio | 0.0763 | 0.1332 | 0.5221 | 0.0886 | 0.1537 | 0.5796 |
| Magdalena | Chibolo | 0.2119 | 0.3698 | 1.4497 | 0.2460 | 0.4269 | 1.6091 |
| Magdalena | Ciénaga | 16.8297 | 29.3744 | 115.1593 | 19.5448 | 33.9087 | 127.8236 |
| Magdalena | Concordia | 0.1264 | 0.2205 | 0.8646 | 0.1467 | 0.2546 | 0.9597 |
| Magdalena | El Banco | 6.2748 | 10.9520 | 42.9360 | 7.2871 | 12.6425 | 47.6578 |
| Magdalena | El Piñón | 1.5637 | 2.7293 | 10.6998 | 1.8160 | 3.1506 | 11.8765 |
| Magdalena | El Retén | 1.1857 | 2.0694 | 8.1130 | 1.3769 | 2.3889 | 9.0052 |
| Magdalena | Fundación | 9.4727 | 16.5336 | 64.8181 | 11.0009 | 19.0857 | 71.9463 |
| Magdalena | Guamal | 2.4458 | 4.2690 | 16.7361 | 2.8404 | 4.9279 | 18.5766 |
| Magdalena | Nueva Granada | 0.5516 | 0.9627 | 3.7741 | 0.6405 | 1.1113 | 4.1891 |
| Magdalena | Pedraza | 0.0285 | 0.0498 | 0.1953 | 0.0331 | 0.0575 | 0.2167 |
| Magdalena | Pijiño del Carmen | 0.5555 | 0.9696 | 3.8012 | 0.6451 | 1.1193 | 4.2193 |
| Magdalena | Pivijay | 2.2499 | 3.9269 | 15.3951 | 2.6129 | 4.5331 | 17.0882 |
| Magdalena | Plato | 1.8045 | 3.1496 | 12.3476 | 2.0956 | 3.6357 | 13.7054 |
| Magdalena | Puebloviejo | 0.5979 | 1.0436 | 4.0912 | 0.6944 | 1.2046 | 4.5411 |
| Magdalena | Remolino | 0.1261 | 0.2201 | 0.8629 | 0.1464 | 0.2541 | 0.9577 |
| Magdalena | Sabanas de San Ángel | 0.7244 | 1.2644 | 4.9571 | 0.8413 | 1.4596 | 5.5023 |
| Magdalena | Salamina | 0.5661 | 0.9880 | 3.8734 | 0.6574 | 1.1405 | 4.2993 |
| Magdalena | San Sebastián de Buenavista | 2.2550 | 3.9358 | 15.4299 | 2.6188 | 4.5433 | 17.1268 |
| Magdalena | San Zenón | 1.0228 | 1.7853 | 6.9990 | 1.1879 | 2.0608 | 7.7686 |
| Magdalena | Santa Ana | 0.6007 | 1.0484 | 4.1102 | 0.6976 | 1.2102 | 4.5622 |
| Magdalena | Santa Bárbara de Pinto | 0.0791 | 0.1381 | 0.5415 | 0.0919 | 0.1594 | 0.6010 |
| Magdalena | Sitionuevo | 2.1532 | 3.7582 | 14.7336 | 2.5006 | 4.3383 | 16.3539 |
| Magdalena | Tenerife | 0.2173 | 0.3792 | 1.4867 | 0.2523 | 0.4378 | 1.6502 |
| Magdalena | Zapayán | 0.0920 | 0.1607 | 0.6298 | 0.1069 | 0.1854 | 0.6991 |
| Magdalena | Zona Bananera | 4.5401 | 7.9243 | 31.0662 | 5.2726 | 9.1475 | 34.4826 |
| Meta | Villavicencio | 44.3916 | 77.4810 | 303.7560 | 51.5534 | 89.4411 | 337.1607 |
| Meta | Acacías | 6.9244 | 12.0858 | 47.3812 | 8.0415 | 13.9514 | 52.5918 |
| Meta | Barranca de Upía | 0.5048 | 0.8810 | 3.4538 | 0.5862 | 1.0170 | 3.8336 |
| Meta | Cabuyaro | 0.2210 | 0.3857 | 1.5121 | 0.2566 | 0.4453 | 1.6784 |
| Meta | Castilla La Nueva | 0.8402 | 1.4665 | 5.7491 | 0.9757 | 1.6928 | 6.3813 |
| Meta | Cubarral | 0.2712 | 0.4733 | 1.8556 | 0.3149 | 0.5464 | 2.0597 |
| Meta | Cumaral | 1.4414 | 2.5158 | 9.8631 | 1.6740 | 2.9042 | 10.9477 |
| Meta | El Calvario | 0.0351 | 0.0612 | 0.2401 | 0.0407 | 0.0707 | 0.2665 |
| Meta | El Castillo | 0.2963 | 0.5171 | 2.0273 | 0.3441 | 0.5969 | 2.2502 |
| Meta | El Dorado | 0.1636 | 0.2855 | 1.1191 | 0.1899 | 0.3295 | 1.2422 |
| Meta | Fuente de Oro | 0.2819 | 0.4921 | 1.9291 | 0.3274 | 0.5680 | 2.1412 |
| Meta | Granada | 6.2198 | 10.8561 | 42.5600 | 7.2233 | 12.5318 | 47.2405 |
| Meta | Guamal | 1.4629 | 2.5533 | 10.0099 | 1.6989 | 2.9474 | 11.1108 |
| Meta | Mapiripán | 0.1144 | 0.1996 | 0.7825 | 0.1328 | 0.2304 | 0.8686 |
| Meta | Mesetas | 0.1540 | 0.2687 | 1.0534 | 0.1788 | 0.3102 | 1.1693 |
| Meta | La Macarena | 0.0688 | 0.1201 | 0.4710 | 0.0799 | 0.1387 | 0.5228 |
| Meta | Uribe | 0.1928 | 0.3365 | 1.3192 | 0.2239 | 0.3884 | 1.4643 |
| Meta | Lejanías | 0.9319 | 1.6265 | 6.3767 | 1.0822 | 1.8776 | 7.0779 |
| Meta | Puerto Concordia | 0.1495 | 0.2609 | 1.0230 | 0.1736 | 0.3012 | 1.1355 |
| Meta | Puerto Gaitán | 6.0356 | 10.5345 | 41.2993 | 7.0093 | 12.1606 | 45.8411 |
| Meta | Puerto López | 2.7202 | 4.7479 | 18.6136 | 3.1591 | 5.4808 | 20.6605 |
| Meta | Puerto Lleras | 0.2169 | 0.3785 | 1.4838 | 0.2518 | 0.4369 | 1.6470 |
| Meta | Puerto Rico | 0.1578 | 0.2755 | 1.0799 | 0.1833 | 0.3180 | 1.1986 |
| Meta | Restrepo | 2.4491 | 4.2747 | 16.7586 | 2.8443 | 4.9346 | 18.6016 |
| Meta | San Carlos de Guaroa | 0.6763 | 1.1804 | 4.6275 | 0.7854 | 1.3626 | 5.1364 |
| Meta | San Juan de Arama | 0.0984 | 0.1718 | 0.6735 | 0.1143 | 0.1983 | 0.7475 |
| Meta | San Juanito | 0.1055 | 0.1841 | 0.7217 | 0.1225 | 0.2125 | 0.8010 |
| Meta | San Martín | 0.7655 | 1.3360 | 5.2378 | 0.8890 | 1.5423 | 5.8138 |
| Meta | Vistahermosa | 0.5833 | 1.0181 | 3.9914 | 0.6774 | 1.1753 | 4.4303 |
| Nariño | Pasto | 28.9717 | 50.5670 | 198.2427 | 33.6457 | 58.3727 | 220.0439 |
| Nariño | Albán | 0.0492 | 0.0859 | 0.3367 | 0.0571 | 0.0991 | 0.3737 |
| Nariño | Aldana | 0.0497 | 0.0868 | 0.3403 | 0.0578 | 0.1002 | 0.3778 |
| Nariño | Ancuyá | 0.0685 | 0.1196 | 0.4689 | 0.0796 | 0.1381 | 0.5205 |
| Nariño | Arboleda | 0.0000 | 0.0000 | 0.0000 | 0.0000 | 0.0000 | 0.0000 |
| Nariño | Barbacoas | 0.3632 | 0.6339 | 2.4850 | 0.4218 | 0.7317 | 2.7583 |
| Nariño | Belén | 0.0000 | 0.0000 | 0.0000 | 0.0000 | 0.0000 | 0.0000 |
| Nariño | Buesaco | 0.1828 | 0.3191 | 1.2509 | 0.2123 | 0.3683 | 1.3884 |
| Nariño | Colón | 0.0293 | 0.0512 | 0.2006 | 0.0340 | 0.0591 | 0.2226 |
| Nariño | Consacá | 0.0640 | 0.1116 | 0.4376 | 0.0743 | 0.1289 | 0.4858 |
| Nariño | Contadero | 0.0301 | 0.0525 | 0.2059 | 0.0349 | 0.0606 | 0.2285 |
| Nariño | Córdoba | 0.1814 | 0.3166 | 1.2411 | 0.2106 | 0.3654 | 1.3776 |
| Nariño | Cuaspud | 0.0074 | 0.0130 | 0.0508 | 0.0086 | 0.0150 | 0.0564 |
| Nariño | Cumbal | 0.1012 | 0.1766 | 0.6923 | 0.1175 | 0.2039 | 0.7685 |
| Nariño | Cumbitara | 0.4507 | 0.7866 | 3.0837 | 0.5234 | 0.9080 | 3.4228 |
| Nariño | Chachagüí | 0.3021 | 0.5273 | 2.0672 | 0.3509 | 0.6087 | 2.2946 |
| Nariño | El Charco | 0.0000 | 0.0000 | 0.0000 | 0.0000 | 0.0000 | 0.0000 |
| Nariño | El Peñol | 0.0012 | 0.0020 | 0.0080 | 0.0014 | 0.0024 | 0.0089 |
| Nariño | El Rosario | 0.2603 | 0.4543 | 1.7810 | 0.3023 | 0.5244 | 1.9768 |
| Nariño | El Tablón de Gómez | 0.0539 | 0.0941 | 0.3689 | 0.0626 | 0.1086 | 0.4095 |
| Nariño | El Tambo | 0.0389 | 0.0679 | 0.2661 | 0.0452 | 0.0784 | 0.2954 |
| Nariño | Funes | 0.0898 | 0.1568 | 0.6147 | 0.1043 | 0.1810 | 0.6823 |
| Nariño | Guachucal | 0.0505 | 0.0882 | 0.3456 | 0.0587 | 0.1018 | 0.3837 |
| Nariño | Guaitarilla | 0.1097 | 0.1915 | 0.7509 | 0.1274 | 0.2211 | 0.8335 |
| Nariño | Gualmatán | 0.0000 | 0.0000 | 0.0000 | 0.0000 | 0.0000 | 0.0000 |
| Nariño | Iles | 0.0000 | 0.0000 | 0.0000 | 0.0000 | 0.0000 | 0.0000 |
| Nariño | Imués | 0.2059 | 0.3594 | 1.4091 | 0.2392 | 0.4149 | 1.5641 |
| Nariño | Ipiales | 20.0710 | 35.0319 | 137.3387 | 23.3091 | 40.4395 | 152.4421 |
| Nariño | La Cruz | 0.1634 | 0.2851 | 1.1178 | 0.1897 | 0.3291 | 1.2407 |
| Nariño | La Florida | 0.0000 | 0.0000 | 0.0000 | 0.0000 | 0.0000 | 0.0000 |
| Nariño | La Llanada | 0.4354 | 0.7599 | 2.9791 | 0.5056 | 0.8772 | 3.3067 |
| Nariño | La Tola | 0.0000 | 0.0000 | 0.0000 | 0.0000 | 0.0000 | 0.0000 |
| Nariño | La Unión | 0.9590 | 1.6738 | 6.5620 | 1.1137 | 1.9322 | 7.2837 |
| Nariño | Leiva | 0.5414 | 0.9449 | 3.7044 | 0.6287 | 1.0908 | 4.1118 |
| Nariño | Linares | 0.1711 | 0.2986 | 1.1708 | 0.1987 | 0.3447 | 1.2995 |
| Nariño | Los Andes | 0.1263 | 0.2204 | 0.8640 | 0.1466 | 0.2544 | 0.9590 |
| Nariño | Magüi | 0.1422 | 0.2483 | 0.9733 | 0.1652 | 0.2866 | 1.0803 |
| Nariño | Mallama | 0.0956 | 0.1668 | 0.6539 | 0.1110 | 0.1926 | 0.7259 |
| Nariño | Mosquera | 0.0013 | 0.0023 | 0.0089 | 0.0015 | 0.0026 | 0.0099 |
| Nariño | Nariño | 1.2139 | 2.1187 | 8.3060 | 1.4097 | 2.4457 | 9.2195 |
| Nariño | Olaya Herrera | 0.1504 | 0.2625 | 1.0291 | 0.1747 | 0.3030 | 1.1423 |
| Nariño | Ospina | 0.0000 | 0.0000 | 0.0000 | 0.0000 | 0.0000 | 0.0000 |
| Nariño | Francisco Pizarro | 0.0000 | 0.0000 | 0.0000 | 0.0000 | 0.0000 | 0.0000 |
| Nariño | Policarpa | 1.0295 | 1.7969 | 7.0447 | 1.1956 | 2.0743 | 7.8194 |
| Nariño | Potosí | 0.1476 | 0.2577 | 1.0103 | 0.1715 | 0.2975 | 1.1214 |
| Nariño | Providencia | 0.1708 | 0.2981 | 1.1686 | 0.1983 | 0.3441 | 1.2971 |
| Nariño | Puerres | 0.2000 | 0.3491 | 1.3687 | 0.2323 | 0.4030 | 1.5192 |
| Nariño | Pupiales | 0.0727 | 0.1269 | 0.4975 | 0.0844 | 0.1465 | 0.5522 |
| Nariño | Ricaurte | 0.2496 | 0.4356 | 1.7079 | 0.2899 | 0.5029 | 1.8957 |
| Nariño | Roberto Payán | 0.0241 | 0.0421 | 0.1651 | 0.0280 | 0.0486 | 0.1832 |
| Nariño | Samaniego | 1.5823 | 2.7618 | 10.8272 | 1.8376 | 3.1881 | 12.0179 |
| Nariño | Sandoná | 0.4406 | 0.7690 | 3.0147 | 0.5117 | 0.8877 | 3.3462 |
| Nariño | San Bernardo | 0.0139 | 0.0242 | 0.0948 | 0.0161 | 0.0279 | 0.1052 |
| Nariño | San Lorenzo | 0.0487 | 0.0850 | 0.3331 | 0.0565 | 0.0981 | 0.3697 |
| Nariño | San Pablo | 0.0509 | 0.0889 | 0.3486 | 0.0592 | 0.1026 | 0.3869 |
| Nariño | San Pedro de Cartago | 0.0000 | 0.0000 | 0.0000 | 0.0000 | 0.0000 | 0.0000 |
| Nariño | Santa Bárbara | 0.0110 | 0.0192 | 0.0755 | 0.0128 | 0.0222 | 0.0838 |
| Nariño | Santacruz | 0.1022 | 0.1784 | 0.6992 | 0.1187 | 0.2059 | 0.7761 |
| Nariño | Taminango | 1.3736 | 2.3974 | 9.3988 | 1.5952 | 2.7675 | 10.4324 |
| Nariño | Tangua | 0.0883 | 0.1542 | 0.6045 | 0.1026 | 0.1780 | 0.6710 |
| Nariño | San Andrés de Tumaco | 7.6643 | 13.3773 | 52.4443 | 8.9008 | 15.4422 | 58.2117 |
| Nariño | Túquerres | 0.7831 | 1.3668 | 5.3586 | 0.9095 | 1.5778 | 5.9479 |
| Nariño | Yacuanquer | 0.3069 | 0.5356 | 2.0997 | 0.3564 | 0.6183 | 2.3307 |
| Norte de Santander | Cúcuta | 341.4569 | 595.9777 | 2,336.4674 | 396.5446 | 687.9743 | 2,593.4140 |
| Norte de Santander | Ábrego | 2.7919 | 4.8730 | 19.1039 | 3.2423 | 5.6252 | 21.2049 |
| Norte de Santander | Arboledas | 0.4558 | 0.7955 | 3.1186 | 0.5293 | 0.9183 | 3.4615 |
| Norte de Santander | Bochalema | 2.0529 | 3.5832 | 14.0476 | 2.3842 | 4.1363 | 15.5924 |
| Norte de Santander | Bucarasica | 0.0992 | 0.1732 | 0.6791 | 0.1153 | 0.2000 | 0.7538 |
| Norte de Santander | Cácota | 0.4461 | 0.7786 | 3.0526 | 0.5181 | 0.8988 | 3.3883 |
| Norte de Santander | Cachirá | 0.1137 | 0.1985 | 0.7781 | 0.1321 | 0.2291 | 0.8637 |
| Norte de Santander | Chinácota | 4.9792 | 8.6907 | 34.0710 | 5.7825 | 10.0322 | 37.8178 |
| Norte de Santander | Chitagá | 2.2077 | 3.8533 | 15.1065 | 2.5639 | 4.4481 | 16.7678 |
| Norte de Santander | Convención | 2.7339 | 4.7718 | 18.7073 | 3.1750 | 5.5084 | 20.7646 |
| Norte de Santander | Cucutilla | 0.2655 | 0.4635 | 1.8170 | 0.3084 | 0.5350 | 2.0168 |
| Norte de Santander | Durania | 0.7986 | 1.3940 | 5.4648 | 0.9275 | 1.6091 | 6.0658 |
| Norte de Santander | El Carmen | 0.4757 | 0.8303 | 3.2549 | 0.5524 | 0.9584 | 3.6129 |
| Norte de Santander | El Tarra | 4.5942 | 8.0186 | 31.4362 | 5.3354 | 9.2564 | 34.8934 |
| Norte de Santander | El Zulia | 9.1074 | 15.8961 | 62.3188 | 10.5767 | 18.3498 | 69.1722 |
| Norte de Santander | Gramalote | 0.3741 | 0.6529 | 2.5598 | 0.4344 | 0.7537 | 2.8413 |
| Norte de Santander | Hacarí | 0.6059 | 1.0576 | 4.1461 | 0.7037 | 1.2208 | 4.6021 |
| Norte de Santander | Herrán | 0.6078 | 1.0608 | 4.1588 | 0.7058 | 1.2245 | 4.6161 |
| Norte de Santander | Labateca | 0.2659 | 0.4641 | 1.8194 | 0.3088 | 0.5357 | 2.0195 |
| Norte de Santander | La Esperanza | 0.2030 | 0.3544 | 1.3892 | 0.2358 | 0.4091 | 1.5420 |
| Norte de Santander | La Playa | 0.1568 | 0.2738 | 1.0732 | 0.1822 | 0.3160 | 1.1913 |
| Norte de Santander | Los Patios | 34.6119 | 60.4115 | 236.8367 | 40.1959 | 69.7367 | 262.8822 |
| Norte de Santander | Lourdes | 0.5478 | 0.9562 | 3.7485 | 0.6362 | 1.1038 | 4.1608 |
| Norte de Santander | Mutiscua | 0.2039 | 0.3559 | 1.3951 | 0.2368 | 0.4108 | 1.5485 |
| Norte de Santander | Ocaña | 31.6812 | 55.2962 | 216.7830 | 36.7923 | 63.8319 | 240.6231 |
| Norte de Santander | Pamplona | 12.1899 | 21.2762 | 83.4110 | 14.1565 | 24.5604 | 92.5839 |
| Norte de Santander | Pamplonita | 0.6904 | 1.2049 | 4.7238 | 0.8017 | 1.3909 | 5.2433 |
| Norte de Santander | Puerto Santander | 6.2542 | 10.9160 | 42.7950 | 7.2632 | 12.6010 | 47.5013 |
| Norte de Santander | Ragonvalia | 1.0407 | 1.8165 | 7.1214 | 1.2086 | 2.0969 | 7.9046 |
| Norte de Santander | Salazar | 1.0237 | 1.7868 | 7.0051 | 1.1889 | 2.0626 | 7.7754 |
| Norte de Santander | San Calixto | 0.8617 | 1.5040 | 5.8961 | 1.0007 | 1.7361 | 6.5445 |
| Norte de Santander | San Cayetano | 1.8649 | 3.2550 | 12.7608 | 2.1658 | 3.7574 | 14.1641 |
| Norte de Santander | Santiago | 0.2293 | 0.4002 | 1.5689 | 0.2663 | 0.4620 | 1.7414 |
| Norte de Santander | Sardinata | 3.8877 | 6.7856 | 26.6024 | 4.5149 | 7.8331 | 29.5279 |
| Norte de Santander | Silos | 0.9069 | 1.5830 | 6.2058 | 1.0533 | 1.8273 | 6.8883 |
| Norte de Santander | Teorama | 1.6380 | 2.8590 | 11.2083 | 1.9023 | 3.3003 | 12.4409 |
| Norte de Santander | Tibú | 18.0859 | 31.5672 | 123.7557 | 21.0038 | 36.4399 | 137.3653 |
| Norte de Santander | Toledo | 1.5054 | 2.6275 | 10.3007 | 1.7482 | 3.0330 | 11.4335 |
| Norte de Santander | Villa Caro | 0.2852 | 0.4977 | 1.9513 | 0.3312 | 0.5746 | 2.1659 |
| Norte de Santander | Villa del Rosario | 83.1483 | 145.1267 | 568.9538 | 96.5627 | 167.5288 | 631.5230 |
| Quindío | Armenia | 44.4470 | 77.5777 | 304.1350 | 51.6177 | 89.5527 | 337.5814 |
| Quindío | Buenavista | 0.1769 | 0.3087 | 1.2101 | 0.2054 | 0.3563 | 1.3432 |
| Quindío | Calarcá | 5.3745 | 9.3807 | 36.7761 | 6.2416 | 10.8287 | 40.8204 |
| Quindío | Circasia | 1.4375 | 2.5089 | 9.8360 | 1.6694 | 2.8962 | 10.9177 |
| Quindío | Córdoba | 0.1634 | 0.2851 | 1.1179 | 0.1897 | 0.3292 | 1.2408 |
| Quindío | Filandia | 1.0181 | 1.7770 | 6.9666 | 1.1824 | 2.0513 | 7.7327 |
| Quindío | Génova | 0.2156 | 0.3764 | 1.4756 | 0.2504 | 0.4345 | 1.6379 |
| Quindío | La Tebaida | 2.6178 | 4.5690 | 17.9124 | 3.0401 | 5.2743 | 19.8823 |
| Quindío | Montenegro | 3.5830 | 6.2537 | 24.5170 | 4.1610 | 7.2191 | 27.2132 |
| Quindío | Pijao | 0.3991 | 0.6965 | 2.7306 | 0.4634 | 0.8040 | 3.0309 |
| Quindío | Quimbaya | 1.9178 | 3.3474 | 13.1230 | 2.2272 | 3.8641 | 14.5662 |
| Quindío | Salento | 1.0654 | 1.8596 | 7.2903 | 1.2373 | 2.1466 | 8.0921 |
| Risaralda | Pereira | 43.0251 | 75.0960 | 294.4057 | 49.9664 | 86.6879 | 326.7822 |
| Risaralda | Apía | 0.3137 | 0.5475 | 2.1465 | 0.3643 | 0.6321 | 2.3826 |
| Risaralda | Balboa | 0.2921 | 0.5099 | 1.9990 | 0.3393 | 0.5886 | 2.2188 |
| Risaralda | Belén de Umbría | 0.6088 | 1.0625 | 4.1655 | 0.7070 | 1.2265 | 4.6236 |
| Risaralda | Dosquebradas | 30.2450 | 52.7896 | 206.9558 | 35.1245 | 60.9383 | 229.7153 |
| Risaralda | Guática | 0.1827 | 0.3189 | 1.2503 | 0.2122 | 0.3681 | 1.3878 |
| Risaralda | La Celia | 0.2840 | 0.4957 | 1.9435 | 0.3298 | 0.5723 | 2.1572 |
| Risaralda | La Virginia | 1.2143 | 2.1195 | 8.3091 | 1.4102 | 2.4466 | 9.2229 |
| Risaralda | Marsella | 0.2942 | 0.5134 | 2.0129 | 0.3416 | 0.5927 | 2.2342 |
| Risaralda | Mistrató | 0.1476 | 0.2577 | 1.0102 | 0.1715 | 0.2975 | 1.1213 |
| Risaralda | Pueblo Rico | 0.0810 | 0.1414 | 0.5545 | 0.0941 | 0.1633 | 0.6155 |
| Risaralda | Quinchía | 0.4913 | 0.8576 | 3.3620 | 0.5706 | 0.9900 | 3.7318 |
| Risaralda | Santa Rosa de Cabal | 4.7962 | 8.3713 | 32.8188 | 5.5700 | 9.6635 | 36.4279 |
| Risaralda | Santuario | 0.3317 | 0.5790 | 2.2700 | 0.3853 | 0.6684 | 2.5196 |
| Santander | Bucaramanga | 106.2693 | 185.4821 | 727.1629 | 123.4139 | 214.1136 | 807.1306 |
| Santander | Aguada | 0.0000 | 0.0000 | 0.0000 | 0.0000 | 0.0000 | 0.0000 |
| Santander | Albania | 0.0000 | 0.0000 | 0.0000 | 0.0000 | 0.0000 | 0.0000 |
| Santander | Aratoca | 0.4946 | 0.8633 | 3.3844 | 0.5744 | 0.9965 | 3.7566 |
| Santander | Barbosa | 3.3357 | 5.8222 | 22.8251 | 3.8739 | 6.7209 | 25.3353 |
| Santander | Barichara | 0.6770 | 1.1816 | 4.6323 | 0.7862 | 1.3640 | 5.1417 |
| Santander | Barrancabermeja | 22.8348 | 39.8558 | 156.2503 | 26.5188 | 46.0080 | 173.4335 |
| Santander | Betulia | 0.2177 | 0.3800 | 1.4897 | 0.2528 | 0.4386 | 1.6535 |
| Santander | Bolívar | 0.0886 | 0.1547 | 0.6063 | 0.1029 | 0.1785 | 0.6730 |
| Santander | Cabrera | 0.0342 | 0.0596 | 0.2337 | 0.0397 | 0.0688 | 0.2595 |
| Santander | California | 0.0816 | 0.1424 | 0.5584 | 0.0948 | 0.1644 | 0.6198 |
| Santander | Capitanejo | 0.2445 | 0.4267 | 1.6728 | 0.2839 | 0.4926 | 1.8568 |
| Santander | Carcasí | 0.2567 | 0.4481 | 1.7567 | 0.2981 | 0.5173 | 1.9499 |
| Santander | Cepitá | 0.0210 | 0.0366 | 0.1434 | 0.0243 | 0.0422 | 0.1592 |
| Santander | Cerrito | 0.6696 | 1.1687 | 4.5819 | 0.7776 | 1.3491 | 5.0858 |
| Santander | Charalá | 0.6329 | 1.1047 | 4.3307 | 0.7350 | 1.2752 | 4.8070 |
| Santander | Charta | 0.0349 | 0.0609 | 0.2388 | 0.0405 | 0.0703 | 0.2651 |
| Santander | Chima | 0.0185 | 0.0323 | 0.1265 | 0.0215 | 0.0372 | 0.1404 |
| Santander | Chipatá | 0.1537 | 0.2682 | 1.0514 | 0.1784 | 0.3096 | 1.1670 |
| Santander | Cimitarra | 1.6632 | 2.9030 | 11.3808 | 1.9315 | 3.3511 | 12.6324 |
| Santander | Concepción | 0.2816 | 0.4915 | 1.9269 | 0.3270 | 0.5674 | 2.1388 |
| Santander | Confines | 0.6002 | 1.0477 | 4.1072 | 0.6971 | 1.2094 | 4.5589 |
| Santander | Contratación | 0.0198 | 0.0346 | 0.1357 | 0.0230 | 0.0400 | 0.1506 |
| Santander | Coromoro | 0.0000 | 0.0000 | 0.0000 | 0.0000 | 0.0000 | 0.0000 |
| Santander | Curití | 0.6397 | 1.1165 | 4.3771 | 0.7429 | 1.2888 | 4.8584 |
| Santander | El Carmen de Chucurí | 0.6967 | 1.2161 | 4.7674 | 0.8091 | 1.4038 | 5.2917 |
| Santander | El Guacamayo | 0.0000 | 0.0000 | 0.0000 | 0.0000 | 0.0000 | 0.0000 |
| Santander | El Peñón | 0.0000 | 0.0000 | 0.0000 | 0.0000 | 0.0000 | 0.0000 |
| Santander | El Playón | 0.5234 | 0.9135 | 3.5814 | 0.6078 | 1.0546 | 3.9753 |
| Santander | Encino | 0.0000 | 0.0000 | 0.0000 | 0.0000 | 0.0000 | 0.0000 |
| Santander | Enciso | 0.0887 | 0.1548 | 0.6067 | 0.1030 | 0.1787 | 0.6735 |
| Santander | Florián | 0.0000 | 0.0000 | 0.0000 | 0.0000 | 0.0000 | 0.0000 |
| Santander | Floridablanca | 33.4852 | 58.4450 | 229.1274 | 38.8874 | 67.4667 | 254.3250 |
| Santander | Galán | 0.1041 | 0.1817 | 0.7125 | 0.1209 | 0.2098 | 0.7908 |
| Santander | Gámbita | 0.0184 | 0.0320 | 0.1256 | 0.0213 | 0.0370 | 0.1394 |
| Santander | Girón | 19.1332 | 33.3950 | 130.9214 | 22.2199 | 38.5499 | 145.3191 |
| Santander | Guaca | 0.3123 | 0.5450 | 2.1367 | 0.3626 | 0.6291 | 2.3716 |
| Santander | Guadalupe | 0.1346 | 0.2349 | 0.9207 | 0.1563 | 0.2711 | 1.0220 |
| Santander | Guapotá | 0.0019 | 0.0032 | 0.0127 | 0.0022 | 0.0037 | 0.0141 |
| Santander | Guavatá | 0.1126 | 0.1965 | 0.7702 | 0.1307 | 0.2268 | 0.8549 |
| Santander | Güepsa | 0.4867 | 0.8495 | 3.3304 | 0.5652 | 0.9807 | 3.6967 |
| Santander | Hato | 0.0155 | 0.0270 | 0.1058 | 0.0180 | 0.0312 | 0.1174 |
| Santander | Jesús María | 0.0000 | 0.0000 | 0.0000 | 0.0000 | 0.0000 | 0.0000 |
| Santander | Jordán | 0.0000 | 0.0000 | 0.0000 | 0.0000 | 0.0000 | 0.0000 |
| Santander | La Belleza | 0.1766 | 0.3082 | 1.2084 | 0.2051 | 0.3558 | 1.3413 |
| Santander | Landázuri | 0.1927 | 0.3363 | 1.3184 | 0.2238 | 0.3882 | 1.4633 |
| Santander | La Paz | 0.0000 | 0.0000 | 0.0000 | 0.0000 | 0.0000 | 0.0000 |
| Santander | Lebrija | 6.0313 | 10.5270 | 41.2700 | 7.0043 | 12.1520 | 45.8085 |
| Santander | Los Santos | 0.8984 | 1.5680 | 6.1471 | 1.0433 | 1.8100 | 6.8232 |
| Santander | Macaravita | 0.0000 | 0.0000 | 0.0000 | 0.0000 | 0.0000 | 0.0000 |
| Santander | Málaga | 1.9832 | 3.4615 | 13.5706 | 2.3032 | 3.9959 | 15.0630 |
| Santander | Matanza | 0.3630 | 0.6335 | 2.4836 | 0.4215 | 0.7313 | 2.7567 |
| Santander | Mogotes | 0.4715 | 0.8230 | 3.2266 | 0.5476 | 0.9501 | 3.5815 |
| Santander | Molagavita | 0.0400 | 0.0698 | 0.2738 | 0.0465 | 0.0806 | 0.3039 |
| Santander | Ocamonte | 0.1864 | 0.3254 | 1.2758 | 0.2165 | 0.3756 | 1.4161 |
| Santander | Oiba | 1.4239 | 2.4852 | 9.7430 | 1.6536 | 2.8688 | 10.8144 |
| Santander | Onzaga | 0.0000 | 0.0000 | 0.0000 | 0.0000 | 0.0000 | 0.0000 |
| Santander | Palmar | 0.0711 | 0.1240 | 0.4863 | 0.0825 | 0.1432 | 0.5398 |
| Santander | Palmas del Socorro | 0.0753 | 0.1314 | 0.5150 | 0.0874 | 0.1516 | 0.5716 |
| Santander | Páramo | 0.6156 | 1.0745 | 4.2123 | 0.7149 | 1.2403 | 4.6756 |
| Santander | Piedecuesta | 18.7180 | 32.6703 | 128.0805 | 21.7378 | 37.7134 | 142.1658 |
| Santander | Pinchote | 0.2313 | 0.4038 | 1.5829 | 0.2687 | 0.4661 | 1.7570 |
| Santander | Puente Nacional | 0.5113 | 0.8923 | 3.4983 | 0.5937 | 1.0301 | 3.8830 |
| Santander | Puerto Parra | 0.2960 | 0.5167 | 2.0255 | 0.3438 | 0.5964 | 2.2483 |
| Santander | Puerto Wilches | 2.0376 | 3.5563 | 13.9423 | 2.3663 | 4.1053 | 15.4755 |
| Santander | Rionegro | 1.0166 | 1.7743 | 6.9560 | 1.1806 | 2.0482 | 7.7210 |
| Santander | Sabana de Torres | 2.8431 | 4.9624 | 19.4546 | 3.3018 | 5.7284 | 21.5940 |
| Santander | San Andrés | 0.1865 | 0.3255 | 1.2760 | 0.2166 | 0.3757 | 1.4163 |
| Santander | San Benito | 0.0000 | 0.0000 | 0.0000 | 0.0000 | 0.0000 | 0.0000 |
| Santander | San Gil | 6.8857 | 12.0183 | 47.1165 | 7.9966 | 13.8735 | 52.2980 |
| Santander | San Joaquín | 0.0012 | 0.0020 | 0.0080 | 0.0014 | 0.0024 | 0.0089 |
| Santander | San José de Miranda | 0.0098 | 0.0170 | 0.0668 | 0.0113 | 0.0197 | 0.0742 |
| Santander | San Miguel | 0.0389 | 0.0679 | 0.2661 | 0.0452 | 0.0783 | 0.2953 |
| Santander | San Vicente de Chucurí | 1.9747 | 3.4466 | 13.5119 | 2.2932 | 3.9786 | 14.9978 |
| Santander | Santa Bárbara | 0.1058 | 0.1846 | 0.7238 | 0.1228 | 0.2131 | 0.8034 |
| Santander | Santa Helena del Opón | 0.0642 | 0.1121 | 0.4396 | 0.0746 | 0.1295 | 0.4880 |
| Santander | Simacota | 0.1586 | 0.2768 | 1.0851 | 0.1842 | 0.3195 | 1.2045 |
| Santander | Socorro | 4.2301 | 7.3831 | 28.9448 | 4.9125 | 8.5228 | 32.1279 |
| Santander | Suaita | 0.7269 | 1.2687 | 4.9737 | 0.8441 | 1.4645 | 5.5207 |
| Santander | Sucre | 0.0263 | 0.0460 | 0.1802 | 0.0306 | 0.0531 | 0.2000 |
| Santander | Suratá | 0.0330 | 0.0576 | 0.2259 | 0.0383 | 0.0665 | 0.2507 |
| Santander | Tona | 0.5886 | 1.0273 | 4.0273 | 0.6835 | 1.1858 | 4.4702 |
| Santander | Valle de San José | 1.6942 | 2.9570 | 11.5926 | 1.9675 | 3.4134 | 12.8675 |
| Santander | Vélez | 0.8314 | 1.4512 | 5.6891 | 0.9656 | 1.6752 | 6.3148 |
| Santander | Vetas | 0.0057 | 0.0099 | 0.0389 | 0.0066 | 0.0115 | 0.0432 |
| Santander | Villanueva | 0.5044 | 0.8804 | 3.4515 | 0.5858 | 1.0163 | 3.8310 |
| Santander | Zapatoca | 0.2334 | 0.4074 | 1.5972 | 0.2711 | 0.4703 | 1.7728 |
| Sucre | Sincelejo | 36.4401 | 63.6024 | 249.3463 | 42.3190 | 73.4202 | 276.7674 |
| Sucre | Buenavista | 0.2104 | 0.3672 | 1.4395 | 0.2443 | 0.4239 | 1.5978 |
| Sucre | Caimito | 0.1440 | 0.2513 | 0.9853 | 0.1672 | 0.2901 | 1.0936 |
| Sucre | Colosó | 0.3157 | 0.5511 | 2.1605 | 0.3667 | 0.6362 | 2.3981 |
| Sucre | Corozal | 3.9586 | 6.9093 | 27.0871 | 4.5972 | 7.9758 | 30.0659 |
| Sucre | Coveñas | 1.7515 | 3.0571 | 11.9851 | 2.0341 | 3.5290 | 13.3032 |
| Sucre | Chalán | 0.0172 | 0.0301 | 0.1179 | 0.0200 | 0.0347 | 0.1309 |
| Sucre | El Roble | 0.2294 | 0.4003 | 1.5694 | 0.2664 | 0.4621 | 1.7420 |
| Sucre | Galeras | 0.5836 | 1.0185 | 3.9930 | 0.6777 | 1.1758 | 4.4322 |
| Sucre | Guaranda | 1.0051 | 1.7542 | 6.8773 | 1.1672 | 2.0250 | 7.6336 |
| Sucre | La Unión | 0.2488 | 0.4342 | 1.7022 | 0.2889 | 0.5012 | 1.8894 |
| Sucre | Los Palmitos | 0.8331 | 1.4541 | 5.7005 | 0.9675 | 1.6785 | 6.3274 |
| Sucre | Majagual | 1.5973 | 2.7879 | 10.9295 | 1.8550 | 3.2182 | 12.1314 |
| Sucre | Morroa | 0.8148 | 1.4222 | 5.5757 | 0.9463 | 1.6418 | 6.1889 |
| Sucre | Ovejas | 0.7998 | 1.3959 | 5.4725 | 0.9288 | 1.6114 | 6.0743 |
| Sucre | Palmito | 0.3040 | 0.5306 | 2.0802 | 0.3530 | 0.6125 | 2.3089 |
| Sucre | Sampués | 1.8373 | 3.2068 | 12.5720 | 2.1337 | 3.7018 | 13.9546 |
| Sucre | San Benito Abad | 0.2142 | 0.3738 | 1.4655 | 0.2487 | 0.4315 | 1.6266 |
| Sucre | San Juan de Betulia | 0.3689 | 0.6439 | 2.5244 | 0.4284 | 0.7433 | 2.8020 |
| Sucre | San Marcos | 1.4238 | 2.4851 | 9.7427 | 1.6535 | 2.8688 | 10.8142 |
| Sucre | San Onofre | 1.7176 | 2.9979 | 11.7531 | 1.9947 | 3.4607 | 13.0456 |
| Sucre | San Pedro | 0.2948 | 0.5146 | 2.0175 | 0.3424 | 0.5940 | 2.2393 |
| Sucre | San Luis de Sincé | 1.1508 | 2.0086 | 7.8745 | 1.3365 | 2.3187 | 8.7405 |
| Sucre | Sucre | 0.1182 | 0.2062 | 0.8085 | 0.1372 | 0.2381 | 0.8974 |
| Sucre | Santiago de Tolú | 0.9459 | 1.6509 | 6.4721 | 1.0984 | 1.9057 | 7.1839 |
| Sucre | Tolú Viejo | 2.0690 | 3.6112 | 14.1573 | 2.4028 | 4.1686 | 15.7142 |
| Tolima | Ibagué | 26.4206 | 46.1145 | 180.7868 | 30.6831 | 53.2328 | 200.6683 |
| Tolima | Alpujarra | 0.0000 | 0.0000 | 0.0000 | 0.0000 | 0.0000 | 0.0000 |
| Tolima | Alvarado | 0.1827 | 0.3188 | 1.2500 | 0.2121 | 0.3681 | 1.3875 |
| Tolima | Ambalema | 0.0250 | 0.0436 | 0.1711 | 0.0290 | 0.0504 | 0.1899 |
| Tolima | Anzoátegui | 0.1227 | 0.2142 | 0.8396 | 0.1425 | 0.2472 | 0.9319 |
| Tolima | Armero | 0.0913 | 0.1593 | 0.6247 | 0.1060 | 0.1839 | 0.6934 |
| Tolima | Ataco | 0.1431 | 0.2497 | 0.9789 | 0.1661 | 0.2882 | 1.0865 |
| Tolima | Cajamarca | 0.5915 | 1.0323 | 4.0471 | 0.6869 | 1.1917 | 4.4922 |
| Tolima | Carmen de Apicalá | 0.8774 | 1.5314 | 6.0036 | 1.0189 | 1.7678 | 6.6639 |
| Tolima | Casabianca | 0.0843 | 0.1471 | 0.5768 | 0.0979 | 0.1698 | 0.6402 |
| Tolima | Chaparral | 0.9453 | 1.6499 | 6.4684 | 1.0978 | 1.9046 | 7.1798 |
| Tolima | Coello | 0.0485 | 0.0846 | 0.3317 | 0.0563 | 0.0977 | 0.3682 |
| Tolima | Coyaima | 0.2858 | 0.4988 | 1.9554 | 0.3319 | 0.5758 | 2.1704 |
| Tolima | Cunday | 0.0567 | 0.0989 | 0.3878 | 0.0658 | 0.1142 | 0.4305 |
| Tolima | Dolores | 0.1377 | 0.2403 | 0.9422 | 0.1599 | 0.2774 | 1.0458 |
| Tolima | Espinal | 3.7088 | 6.4733 | 25.3779 | 4.3071 | 7.4725 | 28.1688 |
| Tolima | Falan | 0.2091 | 0.3649 | 1.4305 | 0.2428 | 0.4212 | 1.5878 |
| Tolima | Flandes | 0.5893 | 1.0286 | 4.0326 | 0.6844 | 1.1874 | 4.4761 |
| Tolima | Fresno | 0.8470 | 1.4784 | 5.7957 | 0.9836 | 1.7066 | 6.4331 |
| Tolima | Guamo | 0.9069 | 1.5828 | 6.2054 | 1.0532 | 1.8272 | 6.8878 |
| Tolima | Herveo | 0.0221 | 0.0385 | 0.1509 | 0.0256 | 0.0444 | 0.1675 |
| Tolima | Honda | 0.2631 | 0.4592 | 1.8001 | 0.3055 | 0.5300 | 1.9981 |
| Tolima | Icononzo | 0.3483 | 0.6079 | 2.3833 | 0.4045 | 0.7018 | 2.6454 |
| Tolima | Lérida | 0.4579 | 0.7993 | 3.1335 | 0.5318 | 0.9227 | 3.4782 |
| Tolima | Líbano | 0.5404 | 0.9433 | 3.6980 | 0.6276 | 1.0889 | 4.1047 |
| Tolima | San Sebastián de Mariquita | 1.9892 | 3.4719 | 13.6111 | 2.3101 | 4.0078 | 15.1079 |
| Tolima | Melgar | 4.9801 | 8.6922 | 34.0768 | 5.7835 | 10.0339 | 37.8243 |
| Tolima | Murillo | 0.0000 | 0.0000 | 0.0000 | 0.0000 | 0.0000 | 0.0000 |
| Tolima | Natagaima | 0.0999 | 0.1743 | 0.6834 | 0.1160 | 0.2012 | 0.7585 |
| Tolima | Ortega | 0.1470 | 0.2565 | 1.0055 | 0.1707 | 0.2961 | 1.1161 |
| Tolima | Palocabildo | 0.2532 | 0.4420 | 1.7327 | 0.2941 | 0.5102 | 1.9232 |
| Tolima | Piedras | 0.0208 | 0.0362 | 0.1420 | 0.0241 | 0.0418 | 0.1576 |
| Tolima | Planadas | 0.5551 | 0.9689 | 3.7986 | 0.6447 | 1.1185 | 4.2163 |
| Tolima | Prado | 0.1221 | 0.2131 | 0.8354 | 0.1418 | 0.2460 | 0.9273 |
| Tolima | Purificación | 0.2328 | 0.4064 | 1.5931 | 0.2704 | 0.4691 | 1.7683 |
| Tolima | Rioblanco | 0.2198 | 0.3837 | 1.5041 | 0.2553 | 0.4429 | 1.6695 |
| Tolima | Roncesvalles | 0.0079 | 0.0138 | 0.0542 | 0.0092 | 0.0159 | 0.0601 |
| Tolima | Rovira | 0.6349 | 1.1082 | 4.3447 | 0.7374 | 1.2793 | 4.8225 |
| Tolima | Saldaña | 0.3246 | 0.5666 | 2.2214 | 0.3770 | 0.6541 | 2.4657 |
| Tolima | San Antonio | 0.1061 | 0.1851 | 0.7257 | 0.1232 | 0.2137 | 0.8056 |
| Tolima | San Luis | 0.3166 | 0.5526 | 2.1665 | 0.3677 | 0.6379 | 2.4048 |
| Tolima | Santa Isabel | 0.1006 | 0.1756 | 0.6884 | 0.1168 | 0.2027 | 0.7642 |
| Tolima | Suárez | 0.0000 | 0.0000 | 0.0000 | 0.0000 | 0.0000 | 0.0000 |
| Tolima | Valle de San Juan | 0.2216 | 0.3868 | 1.5163 | 0.2573 | 0.4465 | 1.6830 |
| Tolima | Venadillo | 0.2170 | 0.3787 | 1.4847 | 0.2520 | 0.4372 | 1.6479 |
| Tolima | Villahermosa | 0.0139 | 0.0242 | 0.0948 | 0.0161 | 0.0279 | 0.1052 |
| Tolima | Villarrica | 0.0898 | 0.1568 | 0.6147 | 0.1043 | 0.1810 | 0.6823 |
| Valle del Cauca | Cali | 295.6624 | 516.0483 | 2,023.1124 | 343.3620 | 595.7067 | 2,245.5986 |
| Valle del Cauca | Alcalá | 1.2131 | 2.1173 | 8.3008 | 1.4088 | 2.4442 | 9.2136 |
| Valle del Cauca | Andalucía | 0.5726 | 0.9994 | 3.9182 | 0.6650 | 1.1537 | 4.3491 |
| Valle del Cauca | Ansermanuevo | 0.2691 | 0.4698 | 1.8417 | 0.3126 | 0.5423 | 2.0442 |
| Valle del Cauca | Argelia | 0.2369 | 0.4135 | 1.6209 | 0.2751 | 0.4773 | 1.7992 |
| Valle del Cauca | Bolívar | 0.2477 | 0.4323 | 1.6947 | 0.2876 | 0.4990 | 1.8810 |
| Valle del Cauca | Buenaventura | 8.4150 | 14.6875 | 57.5809 | 9.7726 | 16.9547 | 63.9132 |
| Valle del Cauca | Guadalajara de Buga | 10.2474 | 17.8857 | 70.1191 | 11.9006 | 20.6466 | 77.8302 |
| Valle del Cauca | Bugalagrande | 0.9565 | 1.6694 | 6.5448 | 1.1108 | 1.9271 | 7.2646 |
| Valle del Cauca | Caicedonia | 1.3396 | 2.3381 | 9.1662 | 1.5557 | 2.6990 | 10.1743 |
| Valle del Cauca | Calima | 2.0891 | 3.6462 | 14.2947 | 2.4261 | 4.2091 | 15.8667 |
| Valle del Cauca | Candelaria | 11.6789 | 20.3844 | 79.9148 | 13.5631 | 23.5309 | 88.7032 |
| Valle del Cauca | Cartago | 7.4454 | 12.9953 | 50.9466 | 8.6466 | 15.0012 | 56.5493 |
| Valle del Cauca | Dagua | 3.2082 | 5.5996 | 21.9528 | 3.7258 | 6.4640 | 24.3670 |
| Valle del Cauca | El Águila | 0.0333 | 0.0580 | 0.2276 | 0.0386 | 0.0670 | 0.2526 |
| Valle del Cauca | El Cairo | 0.2449 | 0.4275 | 1.6759 | 0.2844 | 0.4935 | 1.8602 |
| Valle del Cauca | El Cerrito | 5.9919 | 10.4582 | 41.0001 | 6.9585 | 12.0725 | 45.5090 |
| Valle del Cauca | El Dovio | 0.3084 | 0.5383 | 2.1103 | 0.3582 | 0.6214 | 2.3424 |
| Valle del Cauca | Florida | 6.6826 | 11.6638 | 45.7265 | 7.7607 | 13.4642 | 50.7552 |
| Valle del Cauca | Ginebra | 1.7963 | 3.1353 | 12.2917 | 2.0861 | 3.6193 | 13.6435 |
| Valle del Cauca | Guacarí | 2.2125 | 3.8617 | 15.1395 | 2.5695 | 4.4578 | 16.8044 |
| Valle del Cauca | Jamundí | 24.3561 | 42.5111 | 166.6603 | 28.2855 | 49.0732 | 184.9883 |
| Valle del Cauca | La Cumbre | 1.6929 | 2.9548 | 11.5841 | 1.9660 | 3.4109 | 12.8580 |
| Valle del Cauca | La Unión | 3.0359 | 5.2989 | 20.7739 | 3.5257 | 6.1169 | 23.0584 |
| Valle del Cauca | La Victoria | 0.9051 | 1.5797 | 6.1930 | 1.0511 | 1.8235 | 6.8741 |
| Valle del Cauca | Obando | 0.1033 | 0.1803 | 0.7068 | 0.1200 | 0.2081 | 0.7846 |
| Valle del Cauca | Palmira | 39.0335 | 68.1289 | 267.0922 | 45.3308 | 78.6455 | 296.4649 |
| Valle del Cauca | Pradera | 1.6496 | 2.8793 | 11.2879 | 1.9158 | 3.3237 | 12.5292 |
| Valle del Cauca | Restrepo | 1.3327 | 2.3261 | 9.1191 | 1.5477 | 2.6851 | 10.1219 |
| Valle del Cauca | Riofrío | 0.5714 | 0.9973 | 3.9097 | 0.6635 | 1.1512 | 4.3396 |
| Valle del Cauca | Roldanillo | 2.4921 | 4.3498 | 17.0527 | 2.8942 | 5.0212 | 18.9281 |
| Valle del Cauca | San Pedro | 0.6600 | 1.1519 | 4.5159 | 0.7664 | 1.3297 | 5.0126 |
| Valle del Cauca | Sevilla | 1.7928 | 3.1291 | 12.2672 | 2.0820 | 3.6121 | 13.6162 |
| Valle del Cauca | Toro | 0.3463 | 0.6044 | 2.3694 | 0.4021 | 0.6977 | 2.6300 |
| Valle del Cauca | Trujillo | 0.7938 | 1.3854 | 5.4314 | 0.9218 | 1.5993 | 6.0286 |
| Valle del Cauca | Tuluá | 16.4596 | 28.7286 | 112.6273 | 19.1151 | 33.1632 | 125.0132 |
| Valle del Cauca | Ulloa | 0.1133 | 0.1978 | 0.7756 | 0.1316 | 0.2284 | 0.8609 |
| Valle del Cauca | Versalles | 0.0488 | 0.0851 | 0.3338 | 0.0566 | 0.0983 | 0.3705 |
| Valle del Cauca | Vijes | 1.0171 | 1.7753 | 6.9600 | 1.1812 | 2.0494 | 7.7254 |
| Valle del Cauca | Yotoco | 1.1888 | 2.0749 | 8.1342 | 1.3805 | 2.3951 | 9.0288 |
| Valle del Cauca | Yumbo | 16.4444 | 28.7020 | 112.5233 | 19.0974 | 33.1325 | 124.8977 |
| Valle del Cauca | Zarzal | 2.9999 | 5.2361 | 20.5274 | 3.4839 | 6.0443 | 22.7849 |
| Arauca | Arauca | 76.4512 | 133.4377 | 523.1283 | 88.7852 | 154.0355 | 580.6579 |
| Arauca | Arauquita | 21.1597 | 36.9320 | 144.7880 | 24.5734 | 42.6329 | 160.7106 |
| Arauca | Cravo Norte | 0.8173 | 1.4266 | 5.5928 | 0.9492 | 1.6468 | 6.2079 |
| Arauca | Fortul | 5.4747 | 9.5555 | 37.4615 | 6.3579 | 11.0306 | 41.5812 |
| Arauca | Puerto Rondón | 1.2684 | 2.2139 | 8.6794 | 1.4731 | 2.5557 | 9.6339 |
| Arauca | Saravena | 21.9313 | 38.2789 | 150.0682 | 25.4695 | 44.1877 | 166.5715 |
| Arauca | Tame | 19.6023 | 34.2138 | 134.1316 | 22.7648 | 39.4951 | 148.8824 |
| Casanare | Yopal | 26.3487 | 45.9890 | 180.2949 | 30.5996 | 53.0880 | 200.1224 |
| Casanare | Aguazul | 4.6033 | 8.0346 | 31.4986 | 5.3459 | 9.2748 | 34.9626 |
| Casanare | Chámeza | 0.1110 | 0.1937 | 0.7596 | 0.1289 | 0.2237 | 0.8431 |
| Casanare | Hato Corozal | 1.0707 | 1.8687 | 7.3261 | 1.2434 | 2.1572 | 8.1318 |
| Casanare | La Salina | 0.0000 | 0.0000 | 0.0000 | 0.0000 | 0.0000 | 0.0000 |
| Casanare | Maní | 3.3208 | 5.7962 | 22.7234 | 3.8566 | 6.6909 | 25.2223 |
| Casanare | Monterrey | 2.3379 | 4.0806 | 15.9976 | 2.7151 | 4.7105 | 17.7569 |
| Casanare | Nunchía | 0.2224 | 0.3883 | 1.5221 | 0.2583 | 0.4482 | 1.6895 |
| Casanare | Orocué | 0.3912 | 0.6828 | 2.6770 | 0.4543 | 0.7882 | 2.9713 |
| Casanare | Paz de Ariporo | 6.2338 | 10.8805 | 42.6556 | 7.2395 | 12.5600 | 47.3466 |
| Casanare | Pore | 1.2527 | 2.1864 | 8.5716 | 1.4548 | 2.5239 | 9.5143 |
| Casanare | Recetor | 0.0000 | 0.0000 | 0.0000 | 0.0000 | 0.0000 | 0.0000 |
| Casanare | Sabanalarga | 0.0923 | 0.1612 | 0.6319 | 0.1072 | 0.1861 | 0.7014 |
| Casanare | Sácama | 0.0506 | 0.0883 | 0.3461 | 0.0587 | 0.1019 | 0.3841 |
| Casanare | San Luis de Palenque | 0.4301 | 0.7507 | 2.9430 | 0.4995 | 0.8666 | 3.2666 |
| Casanare | Támara | 0.0624 | 0.1090 | 0.4272 | 0.0725 | 0.1258 | 0.4742 |
| Casanare | Tauramena | 3.3222 | 5.7986 | 22.7328 | 3.8582 | 6.6937 | 25.2328 |
| Casanare | Trinidad | 1.1748 | 2.0505 | 8.0386 | 1.3643 | 2.3670 | 8.9226 |
| Casanare | Villanueva | 5.3069 | 9.2627 | 36.3135 | 6.1631 | 10.6925 | 40.3070 |
| Putumayo | Mocoa | 5.2855 | 9.2254 | 36.1671 | 6.1383 | 10.6494 | 40.1444 |
| Putumayo | Colón | 0.2820 | 0.4922 | 1.9296 | 0.3275 | 0.5682 | 2.1418 |
| Putumayo | Orito | 3.1757 | 5.5429 | 21.7303 | 3.6881 | 6.3985 | 24.1201 |
| Putumayo | Puerto Asís | 6.7872 | 11.8464 | 46.4425 | 7.8822 | 13.6750 | 51.5499 |
| Putumayo | Puerto Caicedo | 1.5533 | 2.7110 | 10.6284 | 1.8038 | 3.1295 | 11.7972 |
| Putumayo | Puerto Guzmán | 0.1906 | 0.3326 | 1.3040 | 0.2213 | 0.3840 | 1.4474 |
| Putumayo | Puerto Leguízamo | 0.2070 | 0.3613 | 1.4166 | 0.2404 | 0.4171 | 1.5723 |
| Putumayo | Sibundoy | 0.9732 | 1.6986 | 6.6590 | 1.1302 | 1.9607 | 7.3913 |
| Putumayo | San Francisco | 0.0044 | 0.0077 | 0.0302 | 0.0051 | 0.0089 | 0.0335 |
| Putumayo | San Miguel | 2.2871 | 3.9919 | 15.6500 | 2.6561 | 4.6081 | 17.3710 |
| Putumayo | Santiago | 0.6022 | 1.0512 | 4.1210 | 0.6994 | 1.2134 | 4.5742 |
| Putumayo | Valle del Guamuez | 4.4779 | 7.8156 | 30.6404 | 5.2003 | 9.0221 | 34.0099 |
| Putumayo | Villagarzón | 1.7517 | 3.0574 | 11.9863 | 2.0343 | 3.5294 | 13.3044 |
| Archipiélago de San Andrés | San Andrés | 0.1262 | 0.2202 | 0.8632 | 0.1465 | 0.2542 | 0.9582 |
| Archipiélago de San Andrés | Providencia | 0.0691 | 0.1206 | 0.4729 | 0.0803 | 0.1392 | 0.5249 |
| Amazonas | Leticia | 0.3063 | 0.5347 | 2.0962 | 0.3558 | 0.6172 | 2.3267 |
| Amazonas | El Encanto | 0.0000 | 0.0000 | 0.0000 | 0.0000 | 0.0000 | 0.0000 |
| Amazonas | La Chorrera | 0.0000 | 0.0000 | 0.0000 | 0.0000 | 0.0000 | 0.0000 |
| Amazonas | Puerto Nariño | 0.0000 | 0.0000 | 0.0000 | 0.0000 | 0.0000 | 0.0000 |
| Guainía | Inírida | 6.5724 | 11.4714 | 44.9726 | 7.6327 | 13.2422 | 49.9183 |
| Guainía | Barranco Minas | 0.0000 | 0.0000 | 0.0000 | 0.0000 | 0.0000 | 0.0000 |
| Guainía | San Felipe | 0.0000 | 0.0000 | 0.0000 | 0.0000 | 0.0000 | 0.0000 |
| Guainía | Puerto Colombia | 0.0000 | 0.0000 | 0.0000 | 0.0000 | 0.0000 | 0.0000 |
| Guainía | Cacahual | 0.0132 | 0.0231 | 0.0906 | 0.0154 | 0.0267 | 0.1005 |
| Guainía | Pana Pana | 0.0000 | 0.0000 | 0.0000 | 0.0000 | 0.0000 | 0.0000 |
| Guainía | Morichal | 0.0072 | 0.0126 | 0.0494 | 0.0084 | 0.0146 | 0.0549 |
| Guaviare | San José del Guaviare | 1.7356 | 3.0293 | 11.8760 | 2.0156 | 3.4969 | 13.1821 |
| Guaviare | Calamar | 0.4274 | 0.7459 | 2.9243 | 0.4963 | 0.8611 | 3.2459 |
| Guaviare | El Retorno | 0.0522 | 0.0911 | 0.3571 | 0.0606 | 0.1052 | 0.3964 |
| Guaviare | Miraflores | 0.0000 | 0.0000 | 0.0000 | 0.0000 | 0.0000 | 0.0000 |
| Vaupés | Mitú | 0.0039 | 0.0068 | 0.0268 | 0.0046 | 0.0079 | 0.0298 |
| Vaupés | Pacoa | 0.0000 | 0.0000 | 0.0000 | 0.0000 | 0.0000 | 0.0000 |
| Vaupés | Taraira | 0.0109 | 0.0190 | 0.0746 | 0.0127 | 0.0220 | 0.0829 |
| Vichada | Puerto Carreño | 13.8867 | 24.2377 | 95.0215 | 16.1270 | 27.9791 | 105.4712 |
| Vichada | La Primavera | 0.2167 | 0.3782 | 1.4827 | 0.2516 | 0.4366 | 1.6457 |
| Vichada | Santa Rosalía | 0.0298 | 0.0521 | 0.2042 | 0.0346 | 0.0601 | 0.2266 |
| Vichada | Cumaribo | 0.0987 | 0.1723 | 0.6757 | 0.1147 | 0.1990 | 0.7500 |
